# A lightweight and robust authentication scheme for the healthcare system using public cloud server

**DOI:** 10.1371/journal.pone.0294429

**Published:** 2024-01-30

**Authors:** Irshad Ahmed Abbasi, Saeed Ullah Jan, Abdulrahman Saad Alqahtani, Adnan Shahid Khan, Fahad Algarni

**Affiliations:** 1 Department of Computer Science, College of Science and Arts Belqarn, University of Bisha, Sabtul-Alaya, Saudi Arabia; 2 Faculty of Computer Science and Information Technology, Universiti Malaysia Sarawak, Kota Samarahan, Malaysia; 3 Higher Education Department of Khyber Pakhtunkhwa at Govt. College Wari Dir Upper, Wari, Khyber Pakhtunkhwa, Pakistan; 4 Department of Computer Science, College of Computing and Information Technology, University of Bisha, Bisha, Saudi Arabia; Jaramogi Oginga Odinga University of Science and Technology, KENYA

## Abstract

Cloud computing is vital in various applications, such as healthcare, transportation, governance, and mobile computing. When using a public cloud server, it is mandatory to be secured from all known threats because a minor attacker’s disturbance severely threatens the whole system. A public cloud server is posed with numerous threats; an adversary can easily enter the server to access sensitive information, especially for the healthcare industry, which offers services to patients, researchers, labs, and hospitals in a flexible way with minimal operational costs. It is challenging to make it a reliable system and ensure the privacy and security of a cloud-enabled healthcare system. In this regard, numerous security mechanisms have been proposed in past decades. These protocols either suffer from replay attacks, are completed in three to four round trips or have maximum computation, which means the security doesn’t balance with performance. Thus, this work uses a fuzzy extractor method to propose a robust security method for a cloud-enabled healthcare system based on Elliptic Curve Cryptography (ECC). The proposed scheme’s security analysis has been examined formally with BAN logic, ROM and ProVerif and informally using pragmatic illustration and different attacks’ discussions. The proposed security mechanism is analyzed in terms of communication and computation costs. Upon comparing the proposed protocol with prior work, it has been demonstrated that our scheme is 33.91% better in communication costs and 35.39% superior to its competitors in computation costs.

## 1. Introduction

The effective handling of stored information gathered from different patients has widely been implemented for research, investigation, and treatment in the healthcare system. This sensitive data is collected with the help of wearable devices embedded inside the human body. The network-enabled devices are connected to the public network over several methods like IEEE 802.15.4 port, IEEE 802.16, WiFi, or WiMAX [1]. The sensor has limited low-power battery storage capacity while performing high computation by generating tremendous output, which requires substantial computing power, massive storage capacity, and real-time processing [2]. For this purpose, a public cloud server offers affordable, flexible, high-performance computing, virtualized storage, and software applications for the healthcare system or patient at home [3]. It is cost-effective, scalable, and available for data-driven pervasive healthcare systems; other service providers can also take benefit by demanding the same high-speed online services from it so that to provide high-quality treatment, effective communication of healthcare personnel with patients, doctors, nurses, pharmacists, and other staff members [4].

A medical information system stored in public cloud services can support the healthcare system for numerous delivery services. This facility is made possible by physiological monitoring devices for patients at home directly or with the doctors at a clinic or in the e-healthcare industry [5]. The public cloud server is mature for the interaction and enhanced sharing of valuable information between various medical institutions, hospital systems, and respective care providers. Such a healthcare system is preferred to reduce costs and make efficient processes, preserving the medical record’s privacy and patient’s identity [6]. Patients’ concerns about losing their privacy are a significant hurdle to adopting cloud-based healthcare systems since they may feel uneasy and lack confidence in the service providers to keep their identities a secret. The transmission of patient information through an insecure internet needs to be secure, and patient privacy is preserved [7]. Thus, a public cloud server’s stored medical information system needs a strong authentication technique to protect accessibility, confidence, and authenticity [8].

Similarly, cloud computing must be implemented to meet the tremendous output generated by numerous IoT in the healthcare system, which requires constant availability and storage [9]. Cloud computing is a potential paradigm in computing that transfers the hardware and software platform to outside service providers (e-healthcare systems) who provide the healthcare facilities to its users (patients) at a significantly lower cost. Cloud computing has much potential for improving the e-healthcare system to ease end-users lives—the cloud transfers patient-sensitive data to healthcare enterprises for management by physicians, labs, research and associated tasks. In general, an e-health cloud is a platform that manages and stores vast amounts of health data from various healthcare providers [10].

Furthermore, attention is required owing to the ubiquitous output of thousands of wearable devices in the healthcare system and the production dispatch for storing it in the public cloud server. As thousands of Internet-of-Medical-Things (IoMT)/sensors generate the result, transmitted to the server for storage requires proper authentication; otherwise, no one builds trust in it due to a lack of privacy, security, and continuous changes in patient conditions which may cause massive mobility issues [11]. All these issues and challenges are due to the need for an appropriate authentication scheme. Therefore, a reliable authentication protocol for such a sensitive environment is much needed to support the accurate authentication of each device, exact data stored in the server, low end-to-end delay, integrity, confidentiality, and low energy consumption. So that, to ensure the privacy of patient security of stored information and deny control of the resources by any fraudulent user. The main contributions of this research work for such a system are as follows:

- An ECC-based lightweight authentication protocol has been proposed to securely provide services to the end-user in the cloud-enabled healthcare system.
- A fuzzy extractor method is used to design the proposed protocol to make the authentication process more secure, and the system shows uniqueness while performing any task. An adversary cannot forge, extract, or collide the hash image generated from user biometrics in combination with a random key extracted before authentication.
- The BAN logic and ROM security proofs were carried out, demonstrating that the proposed protocol is verifiably secure.
- The key secrecy, integrity, confidentiality, and reachability have been verified through a well-known software verification toolkit, ProVerif.

## 2. Literature survey

The growing need and other advancements in computing technology can provide scalable services with the potential to up-size or downsize information storage for cloud computing, which is frequently utilized in the healthcare system. In this regard, Zhang et al. [6] proposed a disease prediction scheme using a cloud server for the healthcare system in which they claimed that security and privacy are two major concerns for such a system. Their strategy was based on the neural network using a single-layer perception (SLP) algorithm containing double layers (input and output). However, their scheme doesn’t fulfil all the security functionalities for participants. Bhatia and Malhotra [7] proposed a scheme based on Morton filters using cloud computing. They claimed that most of the security schemes for patient diagnosis are designed on cuckoo or bloom filters, which suffer from security and privacy issues. However, using their scheme, patients cannot preserve his/her privacy. Sivan and Zukarnain [8] proposed a cloud-based healthcare system using AI (Artificial Intelligence) and ML (Machine Learning) techniques. They addressed privacy and security-related issues and challenges in their research for secure access of patient sensitive information by physicians, labs and hospitals. They also suggested that approximate security solutions can beneficially mitigate all the issues mentioned earlier in the cloud-based healthcare system. Still, they failed to test their methodology in a real-world environment. Chenthara et al. [9] proposed a collaborative mechanism for the healthcare system that can support MPPDS (multi-level privacy-preserving data sharing). However, in some layers, the strong adversary can easily capture data and later be used for replay and DoD attacks.

Huang et al. [10] proposed a security mechanism for MHSN (mobile healthcare social network) based on identity using cloud computing. In their scheme, a user via a mobile device can browse their health information from the cloud and share it with medical professionals for possible diagnosis. However, such a practice cannot be feasible in different countries; their scheme is weaker against MIMT attacks. Li et al. [11] designed a searchable private key cryptographic-based authentication scheme for medical data using cloud computing. Their strategy has dynamically searched a patient’s medical profile in encrypted form over the symmetric key and mitigated the key-sharing drawback. But due to this, the computation costs go high, which in turn doesn’t get the patient’s attention due to the slow computation of patient-sensitive data in the form of X-ray images, ECG, and EEG. Ma et al. [12] proposed a multi-access attribute-based encryption (MA-ABE) scheme for people who desire to restrict their continuous hospital visits for diagnosis. It still doesn’t provide efficient and effective services to the community due to low performance. Nguyen et al. [13] proposed a blockchain-based secure authentication scheme for electronic healthcare records (EHRs). They claimed that their strategy provides low operational costs, availability, and flexibility. However, using cryptographic primitives for blockchain technology can degrade the performance metrics for end users/patients. Chen et al. [14] demonstrated that to preserve patient-sensitive information (privacy), electronic medical records must be secure from intruders. In this regard, they proposed a framework that offered authorization, confidentiality, and integrity of records efficiently. However, on one side, they secure the record, while on the other, their performance degrades. Wu et al. [15] used a simple cryptographic hash function to design a scheme to secure patient information through cloud computing. But a simple hash cryptographic function can provide secure services to a single user; when the number of users/patients increases, their scheme isn’t feasible; therefore, it fails to provide efficient and reliable services. The remaining related literature review in the form of a table is also shown in Table 1.

**Table 1:** Critical literature review.

Ref#	Year	Approach	Advantages	Limitations
**[16]**	2016	Healthcare devices can be connected to the cloud server for numerous tasks.	Excellent performance achieved	However, when the connection is dropped, the outputs generated by the devices are a soft target for attackers.
**[17]**	2016	A symmetric cryptographic algorithm and crypto hash function-based authentication scheme for IoT-based e-healthcare system.	Third-party involvement makes the system credible for integrity, confidentially and availability of physiological parameters.	Rigorously, their scheme can be feasible for two to three parties/IoT; when the number of sensor nodes increases, their scheme doesn’t perform well, and authentication takes maximum computation costs.
**[18]**	2017	Cryptographic Algorithms	Mitigated ambulance incidents, heart attacks, and brain stroke.	The response time is much slower for such sensitive data broadcasting.
**[19]**	2017	A security mechanism that secures the transmission between fog and healthcare devices.	Effectively alleviate the interoperability challenges between cloud and edge computing paradigms.	However, due to the non-usage of the cryptographic approach, their strategy can easily be hacked.
**[20]**	2018	ECC	It efficiently mitigated the stolen verifier, impersonation, and insider attacks.	The user is traceable.
**[21]**	2019	Blockchain	Successfully achieved authentication	Vulnerable to insider and sniffing attacks
**[22]**	2019	Zero-knowledge concept and fuzzy extractor	Successfully segmenting the patient parenchyma of CT lung images	The security analysis section is missing
**[23]**	2019	certificate-less cryptography	Achieved anonymity and privacy	Feasible for only two-party communications.
**[24]**	2019	fuzzy extractor	No one is cracking/guessing the biometrics of a user.	Vulnerable to password-guessing attacks.
**[25]**	2020	MBFTA (mixed Byzantine fault tolerance algorithm)	Efficient transaction handling, anonymity, and privacy are achieved.	Data obfuscation and malicious operations might have yet to be tackled.
**[26]**	2020	Message Authentication Code (MAC) Secure Hash Algorithm (SHA-1)	The researchers have seriously used a one-time biometric key +MAC-SHA1 along with random mapping – which means the adversary doesn’t violate the security features while using their scheme.	However, it is feasible for one-party authentication when the number of nodes/entities increases; their scheme, which is based on MAC-SHA1, cannot be feasible.
**[27]**	2022	Batch authentication algorithm	Balance of security with performance has been seen.	Batch verification of messages is a little bit heavyweight
**[28]**	2023	A biometric-based key validation	The researchers have efficiently utilized the low latency WBAN for deployed cryptographic primitives.	However, prompt data delivery causes disastrous results, potential threats to patient freedom and data consistency issues.
**[29]**	2023	ECC for IoT-enabled cloud server	The researchers have used KeyGen(.), SigGen(.), GenProof(.) and VerifyProof(.) algorithms, which the adversary cannot break the session key during its establishment for secure communication.	However, due to using a smart device to store security credentials, adversaries can easily launch a stolen verifier attack on their scheme.

Therefore, considering the literature survey, it has been observed over the recent years, that different researchers have proposed numerous schemes at different times. These schemes are either based on bilinear pairing, discrete logarithm problems, RSA, or other cryptographic techniques with high communication/computation costs due to exponential execution complexity or suffer from security and privacy issues, most of which are unable to withstand the known vulnerabilities. In this regard, we have proposed a scheme in section 4 of the research paper based on ECC, using fuzzy extractor method that can offer better security and efficient performance then recent schemes.

## 3. Preliminaries

This section will demonstrate the basic concept for designing the proposed authentication schemes. These foundation terminologies are described as under:

### 3.1 System architecture

The architecture aims to secure the cloud-based patient remote monitoring without losing privacy and performance. So, the architecture consisted of tiny sensors affixed to the patient’s body to collect physiological parameters. These network-enabled sensors are connected to an external network or other devices through IEEE 802.15.4 port and then to the main network to enable external connectivity. So far, two participants are involved in the proposed system architecture are public cloud server and patient/end-user. These are described as under:

*Public Cloud Server (PCS)*: A system containing unlimited storage capacity for storing hounds of hundreds of medical data generated by millions of wearable devices embedded inside the human body. Hospitals that desire to operationalize their healthcare system into a cloud-based system and connect numerous wearable devices can outsource physiological data from a public cloud server. The public cloud server can also have strong computation capabilities for the stored sensitive patient information, learning, and diagnosis prediction.

**Fig 1:**
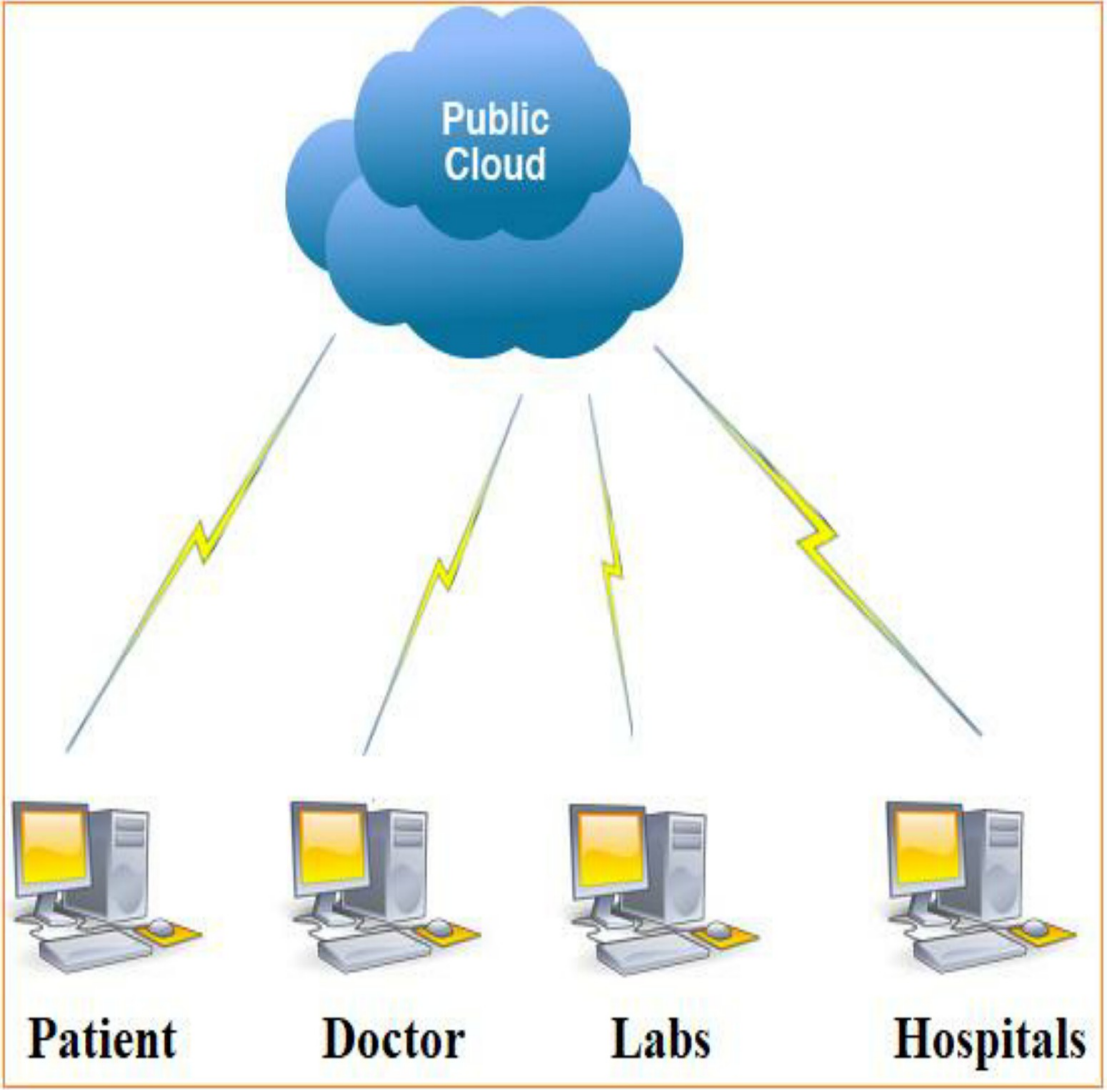
System architecture.

*Patient or User (U)*: A person who accesses the entire system takes medical data from either doctors or embedded sensors and then sent onward to the hospitals for treatment. All patients must trust the healthcare system as it provides identity to the patient and can also be responsible for the diagnosis, treatment, and real-time monitoring. Hospitals must also offer intelligent services to patients to securely check their status and online suggested treatment, disease confirmation, and different symptoms, as shown in Fig 1.

### 3.2 Elliptic Curve Cryptography (ECC)

This type of asymmetric key cryptography or public key cryptography is based on a curve over a finite field of equation defined in the form y^2^=x^3^+ax+b over an F_q_(a, b) (finite field). ECC [30] has the following unique features:

- It is also called NP-problem, which is hard for adversary *A* to break, whereas a, b set of finite field elements on the curve. Let a point in the curve is P, and its base is Q; then *Q=s*.*P* which is computationally difficult to find *s* (integer value), means infeasible.
- If we draw a chord that interests the curve at a third point, the result reflects on the x-axis and is represented by –R.
- It offers more efficient security than RSA.
- By giving points xP, yP, P over F_q_(x, y), it is much more challenging to calculate xyP.
- Let (a, b)∈E and a≠b, then ⇒R=a+b. So according to the curve equation in ECC, x^3^=*λ*^*2*^*-x-x*^*2*^, y^3^=*λ(x+x*^*3*^*)-y whereas λ=(y*^*2*^*-y)/x*^*2*^*-x)*.
- Let a point P is doubled in the curve such that P∈E whereas P≠-P, then ⇒R=2P. So according to ECC, x^3^= *λ*^*2*^*-2x*, *y*^*3*^*= λ(x+x*^*3*^*) whereas λ=(3x*^*2*^*-a)/2y*.
- ECC is an interesting asymmetric approach that offers greater security with a smaller key size than RSA. For example, if the key length in RSA becomes 1024, then the same key will be 160 bits in ECC. Similarly, if the ECC key size is 256 bits, it will be 3072 bits in RSA. The ratio of ECC and RSA is 1:6, meaning ECC is six times smaller than RSA and offers excellent security.

### 3.3 Fuzzy extractor

Universally, academia and industries are using the conventional cryptographically generated key for authentication, which could be a better practice from a security point of view. By considering the more vigorous adversary nature, biometrics in combination with conventional keys are used but using constant iris or fingerprint, which could be a better practice. An adversary can easily forge or duplicate during authentication to collide with the original image/key. Therefore, a fuzzy extractor method is used to make the authentication process more secure, and the system shows uniqueness while performing any task [31].

### 3.4 Threat and adversary model

If adversary *A* obtains the secret session key of a legitimate user/patient and desires to act as a malicious user in spoofing the actual user, adversary *A* plays the role of both active and passive attacker by detecting the exchanged information among the user and server and eavesdropping fake information [32] in the following manner:

1. *A* may disclose/target the session key’s integrity by changing its parameters.
2. *A* might modify the message exchanged publically among the user, server, and vice-versa.
3. *A* might disrupt valuable services among legitimate peers.
4. *A* might gain control of the wireless line/public line for accessing exchanged messages and obtaining its internal secret credential.
5. *A* can also have the power to malfunction the public channel among participants.
6. *A* guess is either the password or ECC key is threatening the confidentiality of the message.
7. *A* can launch a brute force attack, cryptanalyze a scheme, or use a guessing attack to find the right secret login credentials.
8. *A* might discover the secret credential through cryptanalysis technique.
9. *A* can have the power to stop a legitimate user or public cloud server for the established session.
10. *A* exhausts the computation, storage, and other resources to stop working in the communication process.

### 3.5 Review analysis of Azrour et al. scheme

In 2021 Azrour et al. [33] proposed a scheme for remote healthcare authentication through cloud-enabled IoT. They [33] presented their strategy in 5 phases: step, sensor registration, user registration, login & authentication phase, and password update phase. These phases are described one by one as under:

1. ***Setup Phase*:** In this phase, the chief executive of a healthcare system selects a key for public cloud server X_s_, hash h(.), and the server publishes the hash code and keeps it in its memory for future correspondence.
2. ***Sensor Registration Phase***: The server chooses identity for the sensor Id_sn_, unique key K_CS-SNi_, computed SK=h(Id_sn_||K_CS-SNi_), saved Id_sn_ and HSK=SK⊕h(X_s_||Id_sn_) in its memory.
3. ***User Registration Phase*:** The user first selected their identity Id_i_, picked two random numbers, password pw_i_ computed MID=h(Id_i_||a), MPW=h(Id_i_||b) and transmitted {MID, MPW} to server over a secure channel. The server SC picked a random number c, computed V=h(MID||X_s_)⊕h(MPW||c), saved MID, c, and sent V toward the user. The user stored {V, a, b, MID} in its smart card.
4. ***Login & Authentication Phase*:** The user provided their smart card into a terminal and entered Id_i_, pw_i_, the smart card confirming MID=h(Id_i_||a) with the already stored smart card values, if found valid, picked A, computed x=V⊕h(h(Id_i_||pw_i_||b)||c), V_1_=h(x||A) and sent {V_1_, MID, A, Id_sn_, T_1_) towards cloud server. The cloud server checked timestamp T_2_-T_1_≤ΔT, computed w_1_=h(MID||X_s=),_ verified V_1_=h(w_1_||A), picked B, calculated w_2_=HSK⊕h(Id_sn_||X_s_), HID=h(MID||Id_sn_), V_2_=h(HID||w_2_||T_2_||B) and sent {V_2_, B, MID, T_2_} towards sensor over open channel. The checked time stamp T_3_-T_2_≤ΔT, calculated HID^/^=h(MID||Id_sn_), checked V_2_=h(HID^/^||SK||T_2_||B), if found validated, SN picked C, computed V_3_=h(MID||Id_sn_||SK||T_3_||C) and sent {V_3_, C, HID, Id_sn_, T_3_} back towards cloud server over an insecure channel. The SN checked timestamp T_4_-T_3_≤ΔT, confirming V_3_=h(MID||w_3_||T_3_||C), picked D, calculated V_4_=h(w_1_||MID||Id_sn_||T_4_||D), S_key_=h(w_1_||MID||Id_sn_) and sent {V_4_, D, Id_sn_, T_4_) back towards user. The user, too, checked timestamp T_5_-T_4_≤ΔT and confirmed V_4_=h(x||MID||Id_sn_||T_4_||D); if validated, the user too computed S_key_=h(x||MID||Id_sn_) and kept as secret session key.
5. ***Password Update Phase***: The user typed Id_i_, pw_i_, validated MID=h(Id_i_||a) and will be asked to enter a new password pw_i_*, by choosing two numbers a*, b* and computed MID*=h(Id_i_||a*), MPW*=h(Id_i_||pw_i_*||b*) and encrypted the message M_u_=E_SK_(MPW||MPW*||MID||MID*||V) and sent towards the cloud server. The cloud server decrypted using the same key SK M_u_^/^=D_SK_(MPW||MPW*||MID||MID*||V), checked V=h(MID||X_s_)⊕h(MPW||c), if confirmed, the cloud server picked c*, replaced MID, c with MID*, c*, computed V*=h(MID*||X_s_)⊕h(MPW*||c*), M_s_=E_SK_(V*) and sent back to user. The user decrypted M_s_^/^=D_SK_(V*) and replaced V, a, b, MID with V*, a*, b*, MID*.

### 3.6 Weaknesses in Azrour et al. scheme

After the critical review analysis of the Azrour et al. [33] scheme, the following loopholes/weaknesses have been noticed:

1. *Insider Attack*: Due to not using a biometric/fuzzy extractor, an attacker can efficiently compute MID=h(Id_i_||a), x=V⊕h(h(Id_i_||pw_i_||b)||c), and V_1_=h(x||A) by taking any two random numbers. After doing such computations, the attacker enters internally into the server to launch an insider attack.
2. *DoS Attack*: The Id_sn_ is transmitted openly through a public network channel; an attacker can easily intercept the line and copy it for malicious deeds.
3. *Replay Attack*: If an attacker from the open channel captures the identity because it is transmitted openly, they may use it for a potential replay attack at some other time.
4. *Privileged-insider Attack*: In this scheme, in each round trip, a lot of random numbers, like a, b, c, c*, A, B, C, D, and X_s_ have been taken, which a privileged user can use for identifying the real identity of the user, server or sensor and launch personal insider attack for other credential hacking. More explicitly, a privileged user sitting on the system can easily use these numbers to hack the whole system at some other time for other websites or malicious deeds. Here, the researchers have observed that if the system generates numerous random numbers for each session key establishment each time, there is a chance that the system can launch an attack on its own credentials.
5. Finally, in Azrour et al. [33] scheme, the researchers needed to provide the facility for revocation/re-registration.

### 4. Proposed protocol

This section of the article demonstrates the proposed protocol, which consists of the following phases. In contrast, the various notations and descriptions used for designing the protocol are shown in Table 2.

**Table 2:** Notations and their description.

Notation	Description	Notation	Description
U	User	PCS	Public Cloud Server
E(F_q_)	Curve	s	Server secret number
q	Prime Order	O	Base Point of Curve
ID_P_	User Identity	PW_P_	User Password
B_P_	User Biometrics	G(.)	Biometric Generation Function
Rep(.)	Biometric Replication Function	r, r_P_, r_PCS_	Random numbers
a,b	Curve Points	||	Concatenation Function
E_s_(.)	Encryption on s	Dec_s_(.)	Description on s
⊕	XOR Operation	T	Timestamp

### 4.1 Setup phase

Initially, the system creates different parameters for its corresponding participants and then sent according to the requirement. A public cloud server (S_PCS_) first selects a curve E(F_p_) at point p of the base point Os of order prime q. Second, the S_PCS_ chooses hash function h(.), secret number s∈Z*_q_, and computes P_key_=s.O as the public key, stores *s* securely in its memory, and publicize {O, q, E(F_p_). h(.)} parameters.

### 4.2 Registration phase

**Fig 2:**
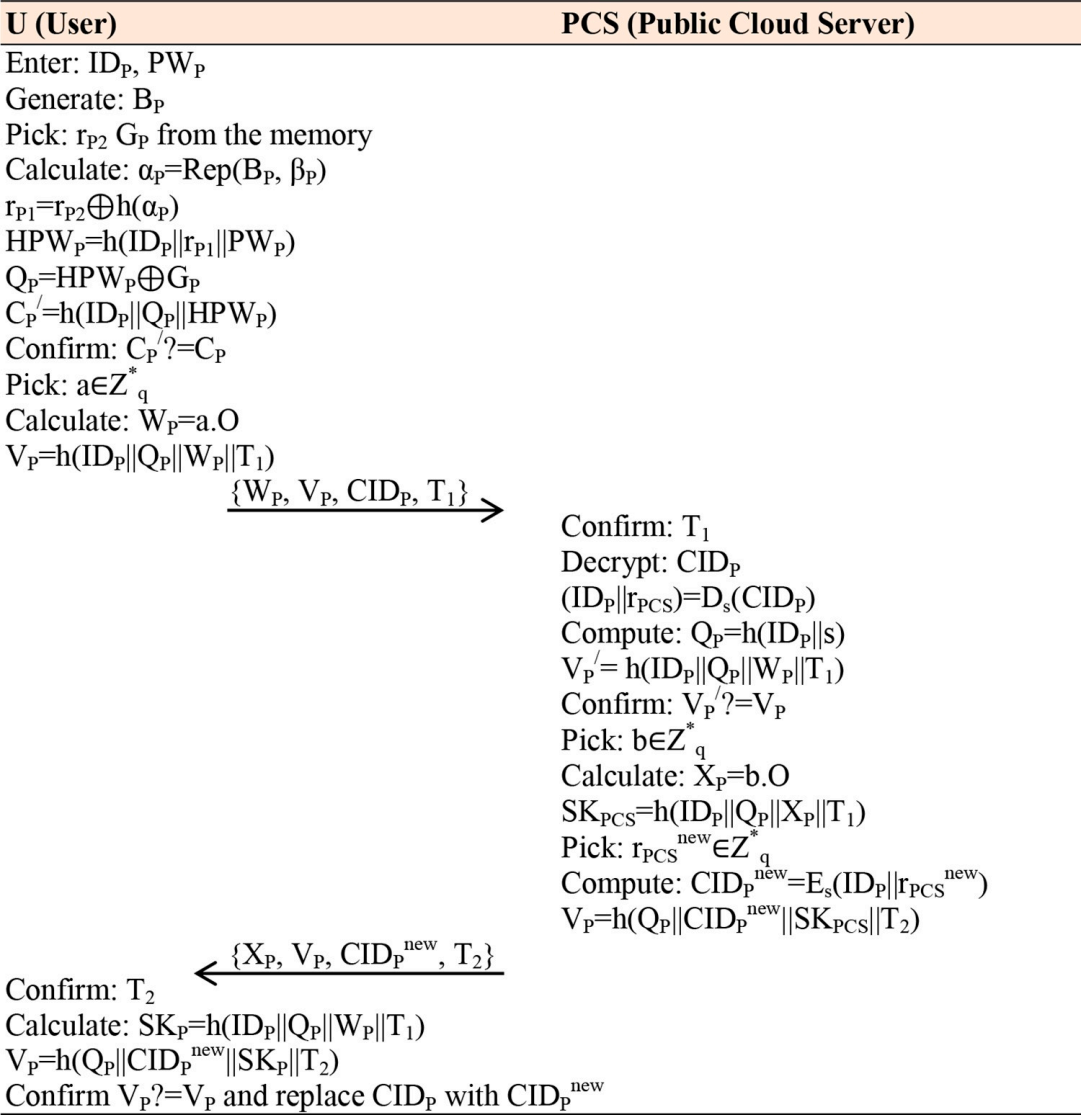
Registration Phase.

In this phase, anyone who desires to access SPCS (Public Cloud Server) must first register with it, and the SPCS provides credentials for future usage. It is worth mentioning that the registration process is mainly accomplished over a private channel in offline mode, which is why open credentials don’t affect the security of a scheme. The following steps will explain it comprehensively:

**Step 1:** The desired user first inputs their identity ID_P_, and password PW_P_ and generates biometrics B_P_. The mobile device fetches r_P1_ and calculates Gen(B_P_)=(α_P_, β_P_), HPW_P_=h(ID_P_||r_P1_||PW_P_) and sends {HPW_P_, ID_P_} message towards the S_PCS_.
**Step 2:** The S_PCS_ confirms the patient’s identity; if it exists, tell them the selection of another unique identity; else, calculates Q_P_=h(ID_P_||s), G_P_=HPW_P_⊕Q_P_, retrieves r_PCS_, computes CID_P_=E_s_(ID_P_||r_PCS_), C_P_=h(ID_P_||Q_P_||HPW_P_), send and stores {CID_P_, G_P_, C_P_, h(.)} in the mobile-device of a patient where it calculates V_P_=r_P_⊕h(α_P_) and keeps {V_P_, β_P_} parameters, as shown in Fig 2.

### 4.3 Authentication phase

**Step 1:** In this protocol’s phase, a legitimate patient enters their identity ID_P_, password PW_P_, and generates B_P_.

**Fig 3:**
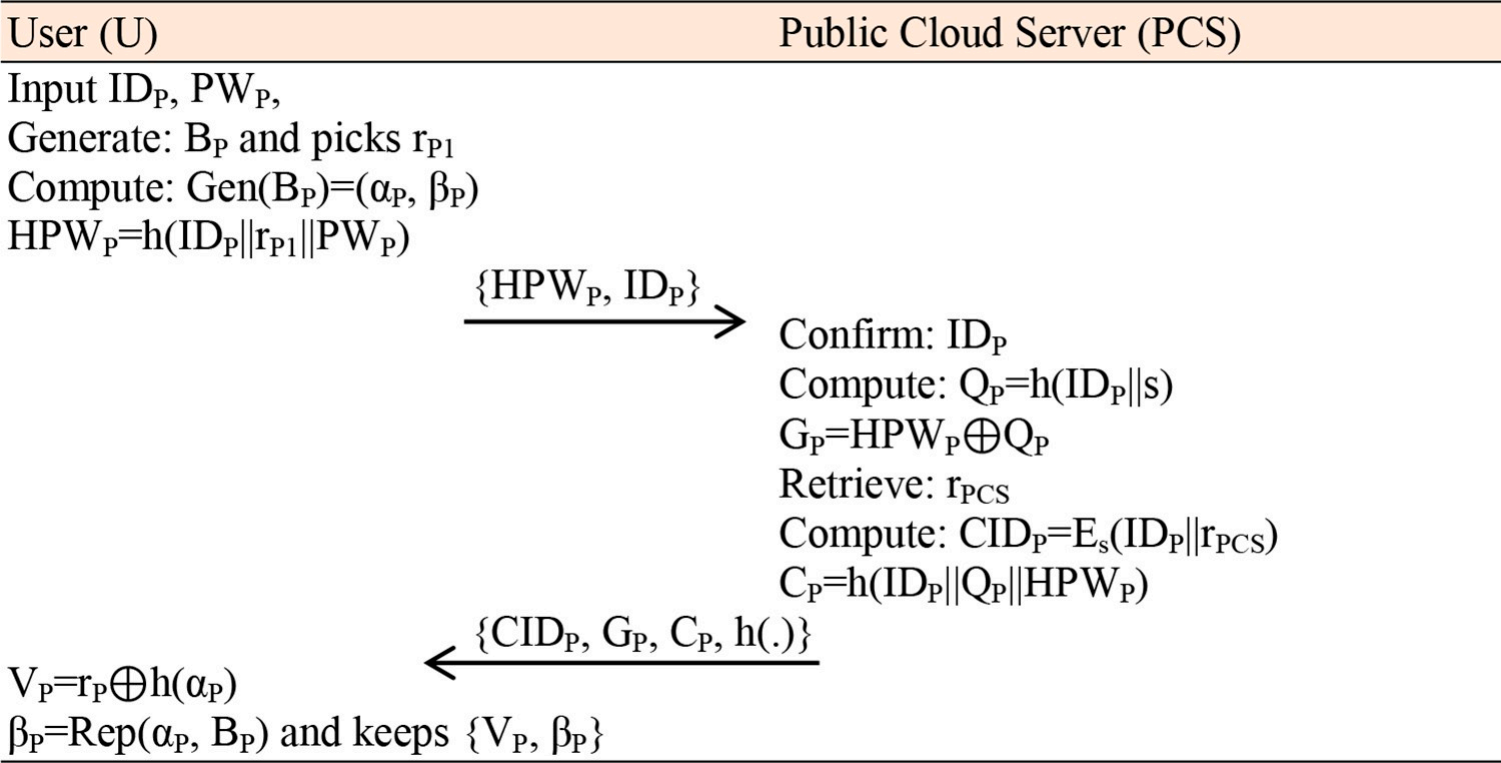
Authentication Phase.

**Step 2:** The mobile device with the patient fetches a random number r_P2_ and G_P_ from memory, calculates α_P_=Rep(B_P_, β_P_), r_P1_=r_P2_⊕h(α_P_), HPW_P_=h(ID_P_||r_P1_||PW_P_), Q_P_=HPW_P_⊕G_P_, C_P_^/^=h(ID_P_||Q_P_||HPW_P_) confirms C_P_^/^?=C_P_. If verified, the mobile device fetches another random number a∈Z*_q_ and calculates W_P_=a.O, V_P_=h(ID_P_||Q_P_||W_P_||T_1_) and transmits {W_P_, V_P_, CID_P_} message towards S_PCS_ over a public channel.
**Step 3:** The S_PCS_ confirms the time and decrypts CID_P_ using the same secret key of S_PCS_, which is (ID_P_||r_PCS_)=D_s_(CID_P_), Q_P_=h(ID_P_||s), V_P_^/^= h(ID_P_||Q_P_||W_P_||T_1_), confirms V_P_^/^?=V_P_, if holds, S_PCS_ fetches another random number b∈Z*_q_, calculates X_P_=b.O, SK_PCS_=h(ID_P_||Q_P_||X_P_||T_1_), create a new number r_PCS_^new^∈Z*_q_, CID_P_^new^=E_s_(ID_P_||r_PCS_^new^), V_P_=h(Q_P_||CID_P_^new^||SK_PCS_||T_2_) and transmits {X_P_, V_P_, CID_P_^new^, T_2_} back towards patient over an open channel.
**Step 4:** The mobile device with the patient calculates SK_P_=h(ID_P_||Q_P_||W_P_||T_1_), V_P_=h(Q_P_||CID_P_^new^||SK_P_||T_2_), confirms V_P_?=V_P_ and replaces CID_P_ with CID_P_^new^, as shown in Fig 3.

### 4.4 Password change phase

In this phase, patients can freely and securely change their passwords and biometrics. In this regard, the patient/user first provides their old ID_P_ and PW_P_ and imprints B_P_ in the application program installed on their mobile. It then calculates α_P_=Rep(B_P_, β_P_), r_P1_=r_P2_⊕h(α_P_), HPW_P_=h(ID_P_||r_P1_||PW_P_), and Q_P_=HPW_P_⊕G_P_. It verifies C_P_?=h(ID_P_||Q_P_||HPW_P_, if confirmed, the patient will be asked to provide a new password PW_P_^new^ and biometrics B_P_^new^. The installed application program will generates r_P_^new^, computes Gen(B_P_^new^)=(α_P_^new^, β_P_^new^), HPW_P_^new^=h(ID_P_||r_P_^new^||PW_P_^new^), G_P_^new^=HPW_P_^new^⊕Q_P_, C_P_^new^=h(ID_P_||Q_P_||HPW_P_^new^), r_P1_=r_P2_⊕h(α_P_^new^) and updates {r_P1_, C_P_, r_P2_, β_P_} with {r_P1_^new^, C_P_^new^, r_P2_^new^, β_P_^new^}.

### 4.5 Patient revocation/re-registration phase

**Fig 4:**
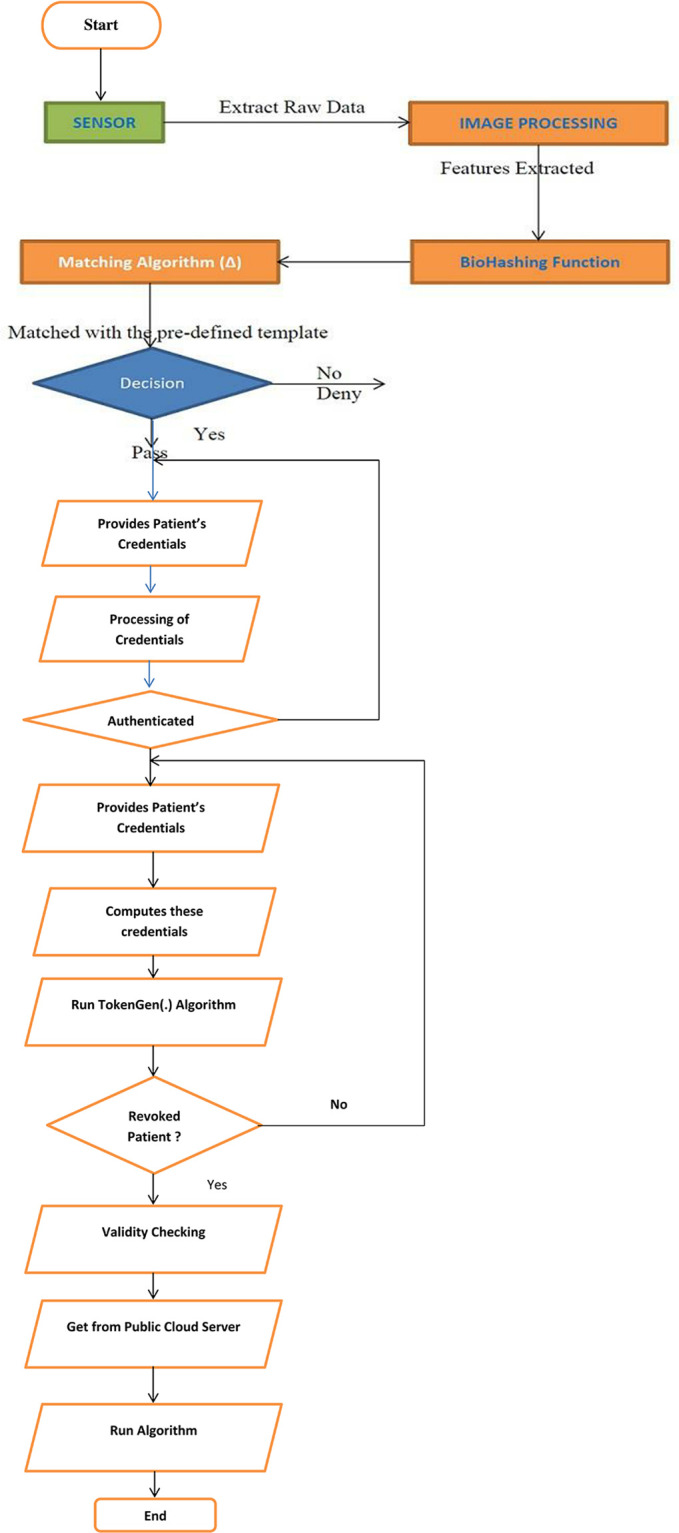
Flow-chart of the whole scenarios.

The proposed authentication protocol provides the facility to revoke/re-issue a patient to/from the public cloud server. In this regard, a patient first verifies their ID_P_, PW_P_, inputs biometrics B_P_, and removes the random number r_P1_ from the records. When the random numbers become removed from the information table and the patient tries to log in from their mobile device, the public cloud server rejects their request because r_P1_ is not available in the record. Similarly, for the re-registration, the patient enters their identity and the public cloud server checks whether it is in the history along with status; if it exists and is in an inactive state, the whole registration phase is executed, the position becomes changed from passive to active by reactivating the patient to the system, as shown in the Fig 4 in the form of flowchart.

## 5 Security analysis

This section will check the protocol’s security using different techniques like BAN logic [34] analysis, ROM [35] analysis, ProVerif [36] simulation, and threats analysis. These are discussed as under:

### 5.1 BAN logic analysis

BAN is a logic of belief was first introduced by three scientists and therefore named as Burrows–Abadi–Needham [34] which is purely used for checking random number reliability, trust, and accuracy in the protocol’s participants. Different notations and rules used in BAN logic are expressed in Table 3, when checking the security of the proposed protocol, we will first express the different rules of BAN logic, define goals, represent idealization and confesses assumptions in achieving the specified goals. These foundations of BAN logic can be explained one by one as under:

**Table 3:** BAN notations and descriptions.

Notation	Description
A |≡ B	Alice A *believe*s in Bob B
A ⇒ B	Alice A has *jurisdiction* over Bob B
A |∼ B	Alice A *once said* Bob B
A ⊲ B	Alice A *sees* Bob B
#(X)	X is said to be *fresh*
<A>_L_	Alice A message is *combined* with formula L
{A}_K_	Alice A message is *encrypted* with key K
(A)_K_	Alice A message is *hashed* with key K

#### i. BAN Logic Rules

BAN rules are comprehensively defined as under:

*Message Meaning*: Suppose Alice A believes in the communication between Alice A and Bob B via a key K and sees a message X combined with key K. In that case, Alice A also believes Bob B has jurisdiction over message X.


$$\frac{A|\equiv A\overset{K}{↔}B⊲{<X>}_{K}}{A|\equiv B|∼X}$$ (1)

*Verification*: If Alice A believes in the freshness of message X, Alice A believes Bob B once said X, then Alice A and Bob B think in message X.


$$\frac{A|\equiv A\overset{K}{↔}B⊲{<X>}_{K}}{A|\equiv B|∼X}$$ (2)

*Freshness*: If Alice A believes in the freshness of message X, Bob B also believes that message X is fresh.

$$\frac{A|\equiv \#(X)}{B|\equiv \#(X)}$$ (3)

*Jurisdiction*: If Alice A and Bob B believe the jurisdiction of message X and Alice A and Bob B once said message X, then Alice A and Bob B think of message X.


$$\frac{A|\equiv \#(X),A|\equiv B|∼X}{A|\equiv B)\equiv X}$$ (4)

#### ii. BAN Logic Goals

BAN logic goals for the proposed scheme are as under:

$$U|\equiv (U\overset{SK}{↔}PCS)$$ (5)

$$U|\equiv S|\equiv (U\overset{SK}{↔}PCS)$$ (6)

$$PCS|\equiv (U\overset{SK}{↔}PCS)$$ (7)

$$PCS|\equiv U|\equiv (U\overset{SK}{↔}PCS)$$ (8)

iii. BAN Logic Idealizations

BAN logic idealizations for the proposed scheme are as under:

$$U\to PCS:\;(IDP,aP):\;(U\overset{h({ID}_{P}||s||T_{1})}{↔}PCS)$$ (9)

$$PCS\to U:\;(bP,U\overset{SK}{↔}PCS):\;(U\overset{h({ID}_{P}|\left|s\right||T_{1})}{↔}PCS)$$ (10)

iv. BAN Logic Assumptions

BAN logic assumptions for the proposed scheme are as under:

U|≡#(aP) (11)

PCS|≡#(bP) (12)

$$U|\equiv (U\overset{h({ID}_{P}||s||T_{1})}{↔}PCS)$$ (13)

$$PCS|\equiv (U\overset{h({ID}_{P}||s||T_{1})}{↔}PCS)$$ (14)

$$U|\equiv PCS|\Rightarrow (U\overset{SK}{↔}PCS)$$ (15)

$$PCS|\equiv U|\Rightarrow (U\overset{SK}{↔}PCS)$$ (16)

v. BAN Logic Proof

BAN logic proof for the proposed scheme is as under:

According to Eq (9), we get

$$U\to PCS:\;(IDP,aP):\;(U\overset{h({ID}_{P}||s||T_{1})}{↔}PCS)$$ (17)

$$PCS⊲(IDP,aP):\;(U\overset{h({ID}_{P}||s||T_{1})}{↔}PCS)$$ (18)

As per Eq (14), we get

$$PCS|\equiv U|∼(IDP,aP)(U\overset{h({ID}_{P}||s||T_{1})}{↔}PCS)$$ (19)

$$PCS|\equiv U|∼(IDP,aP)(U\overset{SK}{↔}PCS)$$ (20)

Looking into Eqs (12) and (2), we get

$$PCS|\equiv U|\equiv (IDP,aP)(U\overset{SK}{↔}PCS)$$ (21)

$$PCS|\equiv U|\equiv (U\overset{SK}{↔}PCS)$$ (22)

G3 Achieved

As per Eq (22), we get

$$PCS|\equiv (U\overset{SK}{↔}PCS)$$ (23)

G4 Achieved

Now, taking Eq (10), we get

$$PCS\to U:\;(bP,U\overset{SK}{↔}PCS):\;(U\overset{h({ID}_{P}|\left|s\right||T_{1})}{↔}PCS)$$ (24)

$$U|\equiv (bP,U\overset{SK}{↔}PCS):\;(U\overset{h({ID}_{P}||s||T_{1})}{↔}PCS)$$ (25)

According to Eqs (24) and (13), we get

$$U|\equiv ((bP,U\overset{SK}{↔}PCS):\;(U\overset{h({ID}_{P}|\left|s\right||T_{1})}{↔}PCS)$$ (26)

$$U|\equiv PCS|∼(bP,U\overset{SK}{↔}PCS)$$ (27)

Eqs (9) and (26), we get

$$U|\equiv PCS|\equiv (U\overset{SK}{↔}PCS)$$ (28)

G2 Achieved

Eq (28) can also be written as:

$$U|\equiv (U\overset{SK}{↔}PCS)$$ (29)

G1 Achieved

BAN logic goals for the proposed scheme are as under:

$$U|\equiv (U\overset{SK}{↔}PCS)$$ (5)

$$U|\equiv S|\equiv (U\overset{SK}{↔}PCS)$$ (6)

$$PCS|\equiv (U\overset{SK}{↔}PCS)$$ (7)

$$PCS|\equiv U|\equiv (U\overset{SK}{↔}PCS)$$ (8)

#### iii. BAN Logic Idealizations

BAN logic idealizations for the proposed scheme are as under:

$$U\to PCS:\;(ID_{P},aP):\;(U\overset{h({ID}_{P}||s||T_{1})}{↔}PCS)$$ (9)

$$PCS\to U:\;(bP,U\overset{SK}{↔}PCS):\;(U\overset{h({ID}_{P}|\left|s\right))T_{1})}{↔}PCS)$$ (10)

#### iv. BAN Logic Assumptions

BAN logic assumptions for the proposed scheme are as under:

U|≡#(aP) (11)

PCS|≡#(bP) (12)

$$U|\equiv (U\overset{h({ID}_{P}||s||T_{1})}{↔}PCS)$$ (13)

$$PCS|\equiv (U\overset{h({ID}_{P}||s||T_{1})}{↔}PCS)$$ (14)

$$U|\equiv PCS|\Rightarrow (U\overset{SK}{↔}PCS)$$ (15)

$$PCS|\equiv U|\Rightarrow (U\overset{SK}{↔}PCS)$$ (16)

#### v. BAN Logic Proof

BAN logic proof for the proposed scheme is as under:

According to Eq (9), we get

$$U\to PCS:\;(ID_{P},aP):\;(U\overset{h({ID}_{P}||s||T_{1})}{↔}PCS)$$ (17)

$$PCS⊲(ID_{P},aP):\;(U\overset{h({ID}_{P}||s||T_{1})}{↔}PCS)$$ (18)

As per Eq (14), we get

$$PCS|\equiv U|∼(ID_{P},aP)(U\overset{h({ID}_{P}||s||T_{1})}{↔}PCS)$$ (19)

$$PCS|\equiv U|∼(ID_{P},aP)(U\overset{SK}{↔}PCS)$$ (20)

Looking into Eqs (12) and (2), we get

$$PCS|\equiv U|\equiv (ID_{P},aP)(U\overset{SK}{↔}PCS)$$ (21)

$$PCS|\equiv U|\equiv (U\overset{SK}{↔}PCS)$$ (22)

G_3_ Achieved

As per Eq (22), we get

$$PCS|\equiv (U\overset{SK}{↔}PCS)$$ (23)

G_4_ Achieved

Now, taking Eq (10), we get

$$PCS\to U:\;(bP,U\overset{SK}{↔}PCS):\;(U\overset{h({ID}_{P}|\left|s\right||T_{1})}{↔}PCS)$$ (24)

$$U|\equiv (bP,U\overset{SK}{↔}PCS):\;(U\overset{h({ID}_{P}||s||T_{1})}{↔}PCS)$$ (25)

According to Eqs (24) and (13), we get

$$U|\equiv ((bP,U\overset{SK}{↔}PCS):\;(U\overset{h({ID}_{P}|\left|s\right||T_{1})}{↔}PCS)$$ (26)

$$U|\equiv PCS|∼(bP,U\overset{SK}{↔}PCS)$$ (27)

Eqs (9) and (26), we get

$$U|\equiv PCS|\equiv (U\overset{SK}{↔}PCS)$$ (28)

G_2_ Achieved

Eq (28) can also be written as:

$$U|\equiv (U\overset{SK}{↔}PCS)$$ (29)

G_1_ Achieved

### 5.2 ROM analysis

In this subsection of the article, we will construct some formal security analysis using Random Oracle Model (ROM) [35] discussions for scrutinizing the ECC key, secret key, and SHA-2 code security. Let adversary *A* breaks the F^SK^ using the following techniques, whereas F^SK^ represents the function session key SK of the user side key computed for establishing a secure session [35].

*Game 1*: In the first step, the adversary attempts to access the public parameters of server λ and is denoted by G_1_ (λ, A), and the answer received is according to like a natural protocol output. Win_0_ represents the winning chance with the adversary.

*Game 2*: The adversary interacts with the protocol by choosing a key K_P_ and updating F^SK-key^. The probability of winning this game with the adversary is *Win*_*1*_ for colliding two ECC keys is:

$$|{Prob}_{A}[{{Win}_{1}(F}^{SK-ECC})]-{Prob}_{A}[{Win}_{0}]|\leq \frac{1}{2^{P(\lambda )}}$$ (30)

*Game 3*: In this game, the key for Enc(.) function is k_1_, the key for the SHA-2 tag is k_2_, and the key SHA-2 tag with reference is checked. Let advantage ADV with A is shown as:

$${|Prob}_{A}[{Win}_{2}(F^{SK-ECC})]-[{Prob}_{A}[{Win}_{1}]|\leq 3{ADV}_{A^{P(\lambda )}}^{KF}$$ (31)

Where KF means key freshness

*Game 4*: In this game, the adversary launches a forgery attack on message m along with the SHA-2 tag and finds a fresh message/SHA-2 tag. The advantage with A to succeed is given as follows:

$$|{Prob}_{A}[{Win}_{3}(F^{SK-ECC})]-[{Prob}_{A}[{Win}_{2}]|\leq {ADV}_{A^{{\left(SHA-2\right)}^{\lambda }}}^{FM}$$ (32)

Where FM means forging a message

*Game 5*: in this game, the Enc(.)/Dec(.) functions made are replaced by an *A* using the k_1_ key and x (the personal values). The advantage with *A* to win this game is:

$$|{Prob}_{A}[{Win}_{4}(F^{SK-ECC})]-[{Prob}_{A}[{Win}_{3}]|\leq {ADV}_{A^{{Enc(.)/Dec(.)}^{\lambda }}}^{Ind}$$ (33)

Where Ind means in-distinguishability

*Game 6*: In this game, the adversary can attempt for the secret key *s* chosen by PCS before executing the protocol. The probability with *A* of guessing the accurate s is:

$$|{Prob}_{A}[{Win}_{5}(F^{SK-s})]-[{Prob}_{A}[{Win}_{4}]|\leq \frac{1}{2^{P(\lambda )}}$$ (34)

*Game 7*: The secret key s_1_ for Enc(.), s_2_ for SHA-2 tag, and s is for execution, then the advantage with *A* is:

$${|Prob}_{A}[{Win}_{6}(F^{SK-s})]-[{Prob}_{A}[{Win}_{5}]|\leq 2{ADV}_{A^{P(\lambda )}}^{KF}$$ (35)

*Game 8*: By launching of forgery attack on secret credentials is performed in this step, and the advantage of winning the game is:

$$|{Prob}_{A}[{Win}_{7}(F^{SK-s})]-[{Prob}_{A}[{Win}_{6}]|\leq {ADV}_{A^{{\left(r\right)}^{\lambda }}}^{FM}$$ (36)

Where r means key randomness and FM means forging personal values.

*Game 9*: By using the secret values, we get

$$|{Prob}_{A}[{Win}_{8}(F^{SK-s})]-[{Prob}_{A}[{Win}_{7}]|\leq {ADV}_{A^{{\frac{Enc(.)}{Dec(.)}}^{\lambda }}}^{ID}$$ (37)

$$|{Prob}_{A}[{Win}_{8}]\leq \left\{ \begin{array}{c} 0\\ 2^{{-p}^{\lambda }} \end{array}\right.$$ (38)

The final chance with *A* of winning the games can be calculated from these games is shown in Eq (39):

$${ADV}_{A}^{SK-ECC}(\lambda ,A)\leq 2\frac{1}{2^{P(\lambda )}}+5{ADV}_{A^{P(\lambda )}}^{SK-s}+2{ADV}_{A^{{\left(SHA-2\right)}^{\lambda }}}^{SK-ECC}+2{ADV}_{A^{{\left(SHA-2\right)}^{\lambda }}}^{SK-ss}$$ (39)

Therefore, considering the above analysis, it has been clear that the in-distinguishability, freshness, and randomness in FSK are verified from Game1 to Game7 [Eqs (30–36), while the confidentiality security feature of the secret values is confirmed from Game8-Game9 [Eqs (37–39)].

### 5.3 ProVerif2.03 simulation

Another method in formal security proof used is to simulate the proposed protocol; in this regard, we have programmed by using a software verification toolkit ProVerif [36]. The ProVerif simulation is a world widely used toolkit for verifying the session key reachability, secrecy, integrity, and confidentiality. Upon running the code, the result shows that the protocol securely exchanges the secret session key among all the participants, and its integrity and reachability have been verified. The result is shown below:

**Table:** 

Completing equations…
-- Process 1-- Query not attacker(SK[]) in process 1
Starting query not attacker(SK[])
RESULT not attacker(SK[]) is true.
Verification summary:
Query not attacker(SK[]) is true.
Query inj-event(end_PCS(IDPCS)) ==> inj-event(start_PCS(IDP)) is true.

### 5.4 Threats analysis

This subsection will scrutinize the scheme’s security by considering prominent attacks like impersonation, online password guessing, stolen-verifier, replay, and offline password guessing attacks. These are described one by one as under:

#### a) Impersonation Attack

The masquerade of a legitimate user logging into a server to avail valuable services, however, in the proposed scheme, let’s suppose someone desires to log in, but they cannot because the procedure is protected in the login by a fuzzy extractor having Gen(.) and Rep(.) functions of the user’s biometrics. Also, the 160-bit long ECC key provides security against illegitimate login attempts. The malicious user cannot enter the server without knowing the password, 160-bit ECC key, and other user credentials. Therefore, an impersonation attack cannot be valid on the proposed scheme.

#### b) Online Password Guessing Attack

If adversary *A* attempts to guess the password by several tries, then A must know r_1_ in the first round to successfully log in to the server. But doing so, *A* cannot guess such a big 160-bit random ECC key; the probability with A is very low for guessing the exact ECC key. Similarly, in the proposed scheme, the password is not retained at the registration phase to be used later for login purposes. The user can set a password and transmit it to the server. If the server observes that someone attempted to enter another password, the server promptly sends a deny message, and the process is discarded. Therefore, the proposed protocol is much safe against online password-guessing attacks.

#### c) Stolen Verifier Attack

The server authenticates anyone from its secret key *s* because no database is there to store the user credentials, so any attempt of an adversary fails. Therefore, the proposed scheme is safe against stolen verifier vulnerability.

#### d) Replay attack

Our scheme will discard an adversary’s potential attempt to replicate an old message due to random checks at each round trip and time threshold. Therefore, this drawback needs to be revised needs to be modified in our scheme.

#### e) Offline Password Guessing Attack

Suppose an attacker obtains the share secrets like ID_P_ r_0_, r_1_, r_2_, and hash codes and later desires to launch an attack on the system by guessing the password in offline mode. However, A cannot succeed due to any knowledge of biometrics, and the fuzzy extractor makes it more unique, so their guessed information cannot compare with the valid parameters. Therefore, the said attempt doesn’t exist in our scheme.

#### f) Forgery Attack

Due to unique session secret key for each session, generation of 160-bit random number from a curve and the presence of timestamp can guarantee forgery attack

#### g) Known session key Attack

The session key is computed from random numbers, timestamp, identities, biometric with fuzzy extractor, no one can launch attack on it. Also if an attacker enter the server and copy the previous session key, he/she cannot identify any credentials from it, as it is computed from different credentials which have been extracted randomly for session key computation.

#### h) Man-in-the-Middle (MITM) Attack

Suppose an adversary *A* intercepts the public channel which the patient communicate his/her information to the cloud server and replaces/modifies/delete/updates the message, in the proposed protocol A cannot calculate V_P_=h(ID_P_||Q_P_||W_P_||T_1_) which is obtained from Q_P_=HPW_P_⊕G_P_. Without knowing Q_P_=HPW_P_⊕G_P_, HPW_P_ and G_P_ adversary cannot reached for server key *s*. Similarly, it is also impossible for A to exactly computes V_P_=h(Q_P_||CID_P_^new^||SK_PCS_||T_2_) because A doesn’t know Q_P_ and other necessary credentials. Also for launching MIMT attack, A must passed V_P_^/^= h(ID_P_||Q_P_||W_P_||T_1_) which is computationally infeasible for him to succeeded for the validation of patient and cloud server. Therefore, our security mechanism is must safe against MIMT attack.

#### i) Privileged Insider Attack

In the registration phase of the proposed protocol, the password sent is HPW_P_=h(ID_P_||r_P1_||PW_P_) in which a privileged user cannot identify because r_p1_ is 160-bit large random point of a curve, unknown to the server as well as the user therefore, our scheme is safe against privileged insider attack.

#### j) DoS Attack

As each round trip of the protocol consisted of a pre-defined time threshold, random checks and 160-bit large random numbers, the DoS attack did not succeed for our scheme. Because when an attacker can copy a message from the open channel, he/she cannot figure out proper credentials from it because the same message in the upcoming round is entirely different due to strong biometrics with fuzzy extractor, ECC key and timestamp. Therefore, our scheme is safe against DoS attacks. Any illegal attempt can automatically be captured due to random checks at each proposed security mechanism round trip.

## 6 Performance analysis

In this section, the performance and comparison analysis of the proposed scheme can be measured by considering storage overheads, communication, and computation costs analysis. These performance metrics are described one by one as under:

### 6.1 Storage overheads analysis

The parameters stored in the registration phase of the proposed scheme are used to calculate the storage overheads of this scheme. So, according to JPBC2.0.0 [37], a 64-bit operating system namely Windows 10, core i_5_ CPU having 8GB of RAM, according to [37, 38], the computation cost of ECC key is 160-bits, hash functions 256-bits, biometric Gen (.) & Rep (.) functions occupy 128-bit space, encryption/decryption functions 192-bits; then the storage costs/overheads of the proposed scheme are shown in Table 4.

**Table 4:** Storage overheads analysis.

Participant	Parameters	Values in Bits	Total Overheads in Bits
User (U)	ID_P_, PW_P_, Gen(.), V_P_, β_P_, r	64+32+128+416+256+160	1056
Server (PCS)	O, q, E(Fp). h(.), s, r_PCS_	160+160+160+256+256+160	1152
**G/Total Storage Costs**			**2208 Bits**

### 6.2 Communication costs

The message exchanged during the authentication phase of the scheme is counted to be the communication costs of a protocol; keeping in view [39], the said costs/overheads of the proposed method are shown in Table 5.

**Table 5:** Communication costs analysis.

Participants	Message	Values in Bits	Total Costs in Bits
U→PCS	{W_P_, V_P_, CID_P_, T_1_}	256+160+192+56	664
PCS→U	{X_P_, V_P_, CID_P_^new^, T_2_}	160+256+192+56	664
**G/Total Communication Costs**			

### 6.3 Computation costs

The time consumed while performing different operations of computation is the computation cost. According to [39], the proposed scheme, let’s suppose T_H_ represents the time of hash function, T_ECC_ describes the extraction of the ECC key, T_XOR_ is the XOR computation time, and T_E/D_ is the time required to compute encryption/decryption functions. The overall computation costs for the proposed scheme are shown in Table 6. It is to mention that the computation costs are considered only for the authentication phase of any authentication scheme; therefore, according to [39], suppose T_ECC_ is 19.2 ms, T_H_ is 0.32 ms, T_XOR_ is negligible equal to zero, and T_E/D_ is 5.6 ms, then the computation costs for the proposed scheme is given as under:

**Table 6:** Computation costs analysis.

Phase	Peer	Operations	Total
Registration	U	1T_ECC_+2T_H_+1T_XOR_+0T_E/D_	3T_ECC_+10T_H_+1T_XOR_+3T_E/D_ 3(19.2)+10(0.32)+0+3(5.6) 57.6+3.2+16.8 77.6 ms
	PCS	1T_ECC_+2T_H_+1T_XOR_+1T_E/D_	
Authentication	U	1T_ECC_+6T_H_+1T_XOR_+1T_E/D_	
	PCS	2T_ECC_+4T_H_+0T_XOR_+2T_E/D_	
Password Change	U	2T_ECC_+3T_H_+4T_XOR_+0T_E/D_	
	PCS	2T_ECC_+5T_H_+3T_XOR_+0T_E/D_	

### 6.4 Comparison analysis

**Fig 5:**
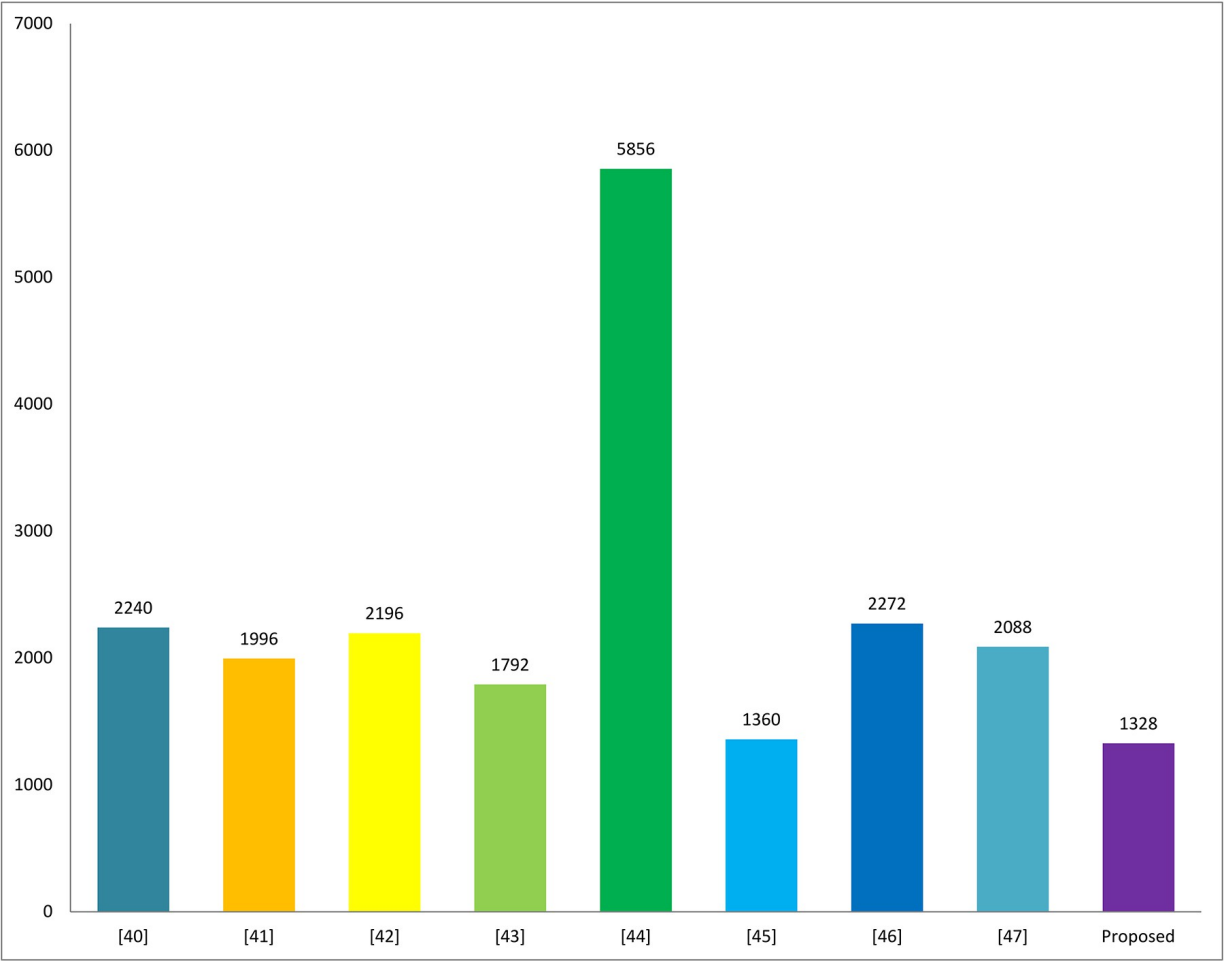
Communication costs comparison.

**Fig 6:**
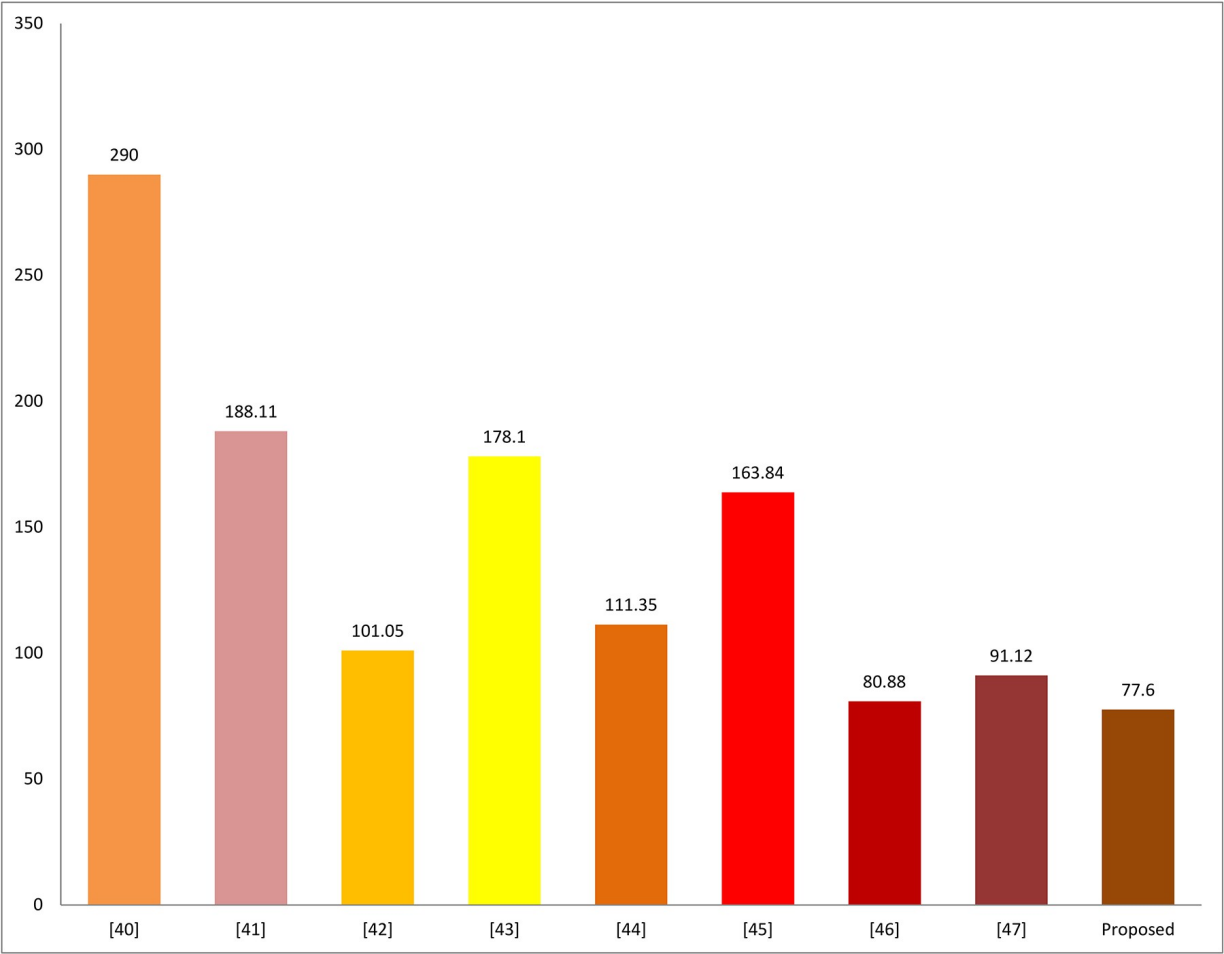
Computation costs comparison.

In this sub-section, we comparatively analyze the proposed protocol regarding the security goals defined in section 2 of the paper with [40–47], as shown in Table 7. Similarly, the proposed scheme can also be compared with [40–47] in terms of communication costs and computation costs are shown in Figs 5 and 6, so that to check the balance of security with performance, both are necessary to be balanced. If one of them is improved, and the other does not, it means that the work couldn’t implement practically for the said sensitive environment, as shown in Table 8 below:

**Table 7:** Comparison analysis in terms of communication costs.

Schemes Performance Metrics↓	[40]	[41]	[42]	[43]	[44]	[45]	[46]	[47]	Proposed
Communication Costs in Bits	2240	1996	2196	1792	5856	1360	2272	2088	1328
Computation Costs in ms	290.00	188.11	101.05	178.10	111.35	163.84	080.88	091.12	077.60

**Table 8:** Comparison analysis in terms of security features.

Goal	Description	[40]	[41]	[42]	[43]	[44]	[45]	[46]	[47]	Proposed
Goal1	De-synchronization attack	N	N	N	N	Y	N	N	Y	N
Goal2	Mutual Authentication	Y	Y	Y	Y	Y	Y	Y	Y	Y
Goal3	spoofing attack	N	Y	N	N	N	N	Y	N	N
Goal4	insider attack	N	N	Y	Y	Y	N	N	N	N
Goal5	Prevention of false messaging	Y	Y	N	Y	Y	Y	Y	Y	Y
Goal6	Anonymity	N	Y	Y	Y	Y	N	Y	Y	Y
Goal7	Accountability	N	N	Y	N	N	N	N	Y	Y
Goal8	replay attack	N	N	N	N	N	N	N	N	N
Goal9	Impersonation attack	N	N	N	Y	N	N	N	N	N
Goal10	perfect forward secrecy	Y	Y	Y	N	Y	Y	Y	Y	Y
Goal11	un-traceability	N	Y	Y	Y	N	N	Y	N	N
Goal12	ESL Attack	N	Y	Y	N	N	N	Y	N	N

**Table 9:** Percentage (%) improvement in performance metrics of the proposed protocol.

The percentage difference between communication and computation costs = $\left(\frac{Comm.Cost–Comp.Cost)}{\frac{\left(Comm.Cost+Comp.Cost\right)}{2}}\right)\times 100$
$=\left(\frac{\left(1360–1328\right)}{\frac{\left(1360+1328\right)}{2}}\right)\times 100\;=\left(\frac{\left(80.88-77.60\right)}{\frac{\left(80.88+77.60\right)}{2}}\right)\times 100$
$=\left(\frac{32}{\frac{2688}{2}}\right)\times 100\;=\left(\frac{3.28}{\frac{2688}{2}}\right)\times 100$
$=\left(\frac{32\times 2}{2688}\right)\times 100\;=\left(\frac{3.28\times 2}{158.48}\right)\times 100$
$=\left(\frac{64}{2688}\right)\times 100\;=\left(\frac{6.56}{158.48}\right)\times 100$
= 2.38% = 4.13%
Which means that our scheme is 2.38% smaller (lightweight) Which means that our scheme is 4.13% is speedy
in size in term of communication overheads. in computation than their competitors.
Keeping in view

Keeping in view the aforementioned percentage difference (Table 9), our scheme is 40.71% better than [40], 33.46% than [41], 39.52% than [42], 77.32% than [43], 2.38% than [45], 41.54% than [46], and 36.39% than [47]. Compared to all, our scheme is 33.91% better in communication costs.

Similarly, for computation costs our scheme is 73.24% superior in computation than [40], 58.74% than [41], 23.20% than [42], 56.42% than [43], 30.30% than [44], 52.63% than [45], 4.13% than [46] and 14.83% than [47]. In average, compared to all, in terms of computation, our scheme takes 35.39% less CPU time than its competitors.

## 7. Conclusion

The patient-sensitive information broadcasting over a public network channel is unsafe as the wireless channels are exposed to various security threats and need protection from multiple attacks. Such a susceptible environment cannot be protected without proper authentication of the end-user, cloud server, and data stored in the server obviously needs a lightweight, robust, and efficient authentication scheme to secure the stored credential and confirm end-user privacy. Therefore, this work provides a method/authentication scheme based on ECC using fuzzy extractor methods. The biometrics generated by the end-users have the capabilities of minimum loss of character and perfect uniqueness due to the fuzzy extractor method. The security of the proposed scheme has been tackled using BAN logic, ROM analysis, ProVerif2.03 simulation, and attack analysis. While in the performance analysis section, we have considered storage, computation, and communication overheads. The result in the comparative analysis section shows that the proposed security mechanism is lightweight and robust to its competitors and can be recommended for a real-world cloud-based healthcare system.

In the future, we plan to develop a secure system using the Cyber Shave Chaotic map method – a double-layer security method in which we will use cryptography in the first layer and steganography in the second layer to protect patient-sensitive information.

## Supporting information

S1 Appendix ProVerif2.03 code. (TXT)

S1 File Figures. (PDF)

S2 File Author biography. (DOCX)
