# Correction: A lightweight and robust authentication scheme for the healthcare system using public cloud server

**DOI:** 10.1371/journal.pone.0318975

**Published:** 2025-02-04

**Authors:** The PLOS ONE Staff

In the Registration phase subsection of the Proposed protocol, there is an error in the first sentence of the first paragraph. The correct sentence is: In this phase, anyone who desires to access S_PCS_ (Public Cloud Server) must first register with it, and the S_PCS_ provides credentials for future usage.

The images for Figs 2 and 3 are incorrectly switched. The image that appears as Fig 2 should be Fig 3, and the image that appears as Fig 3 should be Fig 2. The figure captions appear in the correct order.

**Fig 2:**
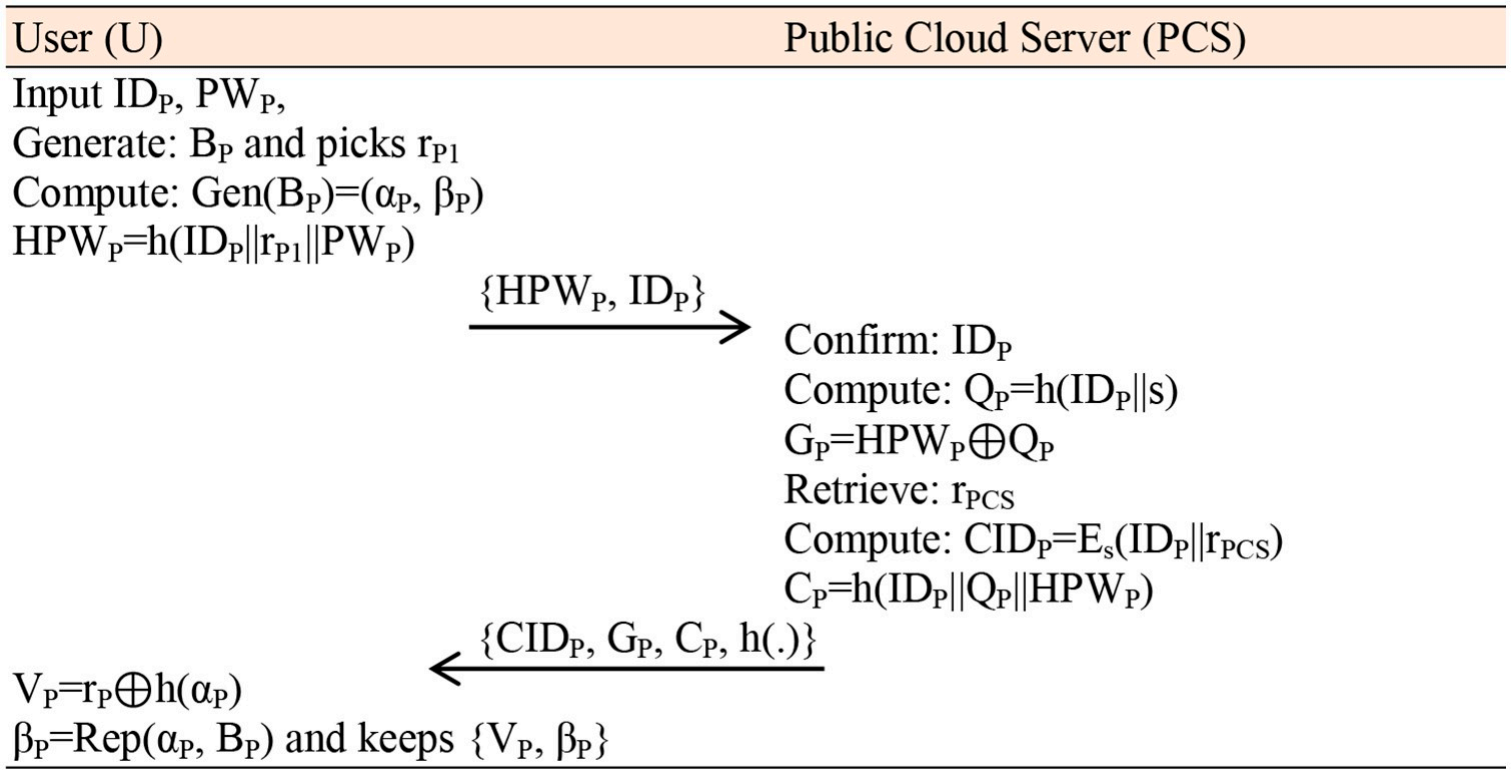
Registration Phase.

**Fig 3:**
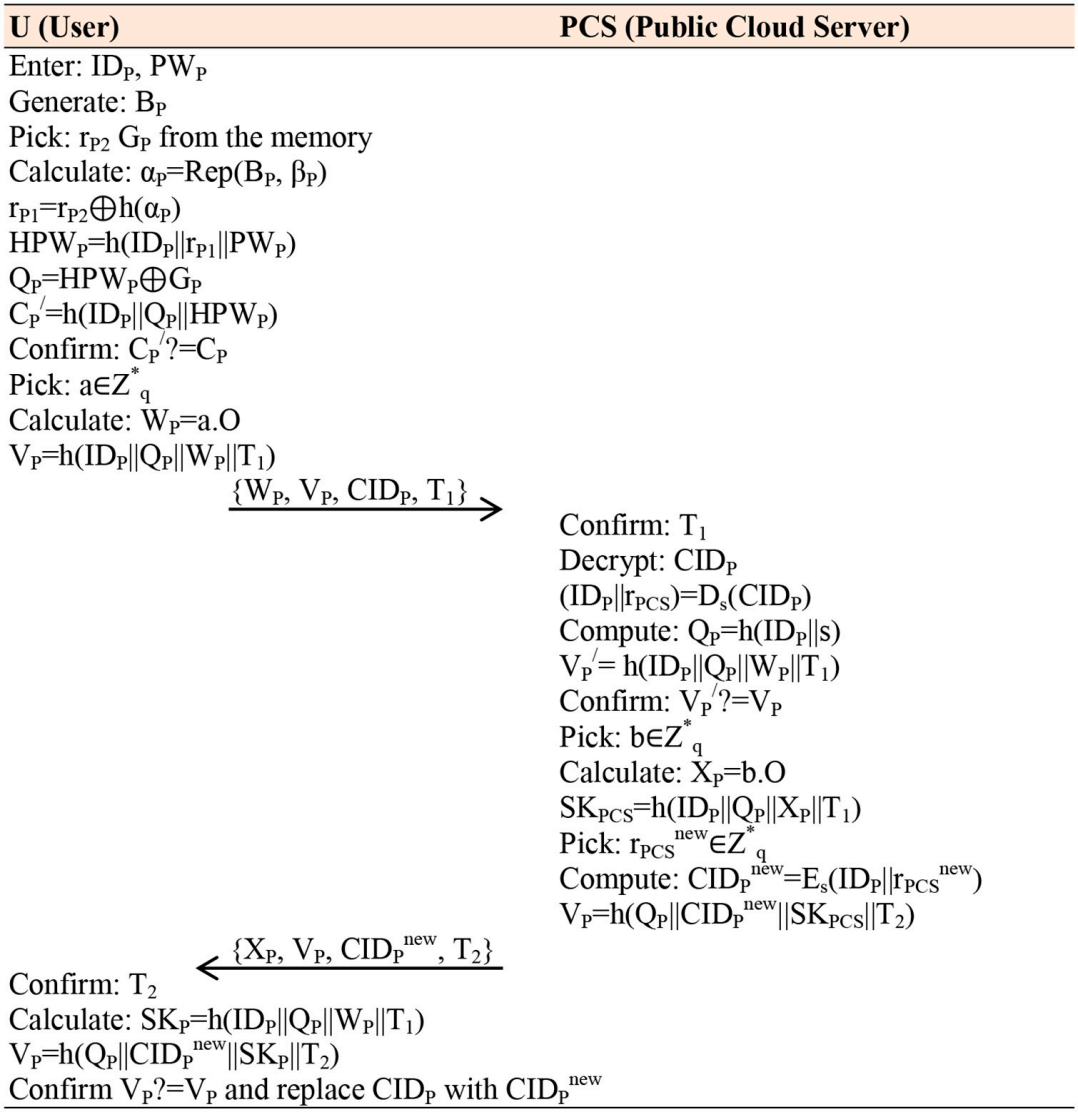
Authentication Phase.

Table 9 is uploaded incorrectly. Please see the correct Table 9 here.

**Table 9:** Percentage (%) improvement in performance metrics of the proposed protocol.

Schemes →	[40]	[41]	[42]	[43]	[44]	[45]	[46]	[47]
% age Improvement of the Proposed protocol in Communication Costs	**40.71%**	**33.46%**	**39.52%**	**25.89%**	**77.32%**	**2.35%**	**41.54%**	**36.39%**
% age Improvement of the Proposed protocol in Computation Costs	**73.24%**	**58.74%**	**23.20%**	**56.42%**	**30.30%**	**52.63%**	**4.13%**	**14.83%**

The publisher apologizes for the errors.
