# Supplementary material for: A lightweight and robust authentication scheme for the healthcare system using public cloud server
Source: PLoS One. 2024 Jan 30;19(1):e0294429. doi: 10.1371/journal.pone.0294429 (PMC10826970; doi:10.1371/journal.pone.0294429)
Supplement: S1 File — (PDF) [file pone.0294429.s002.pdf]

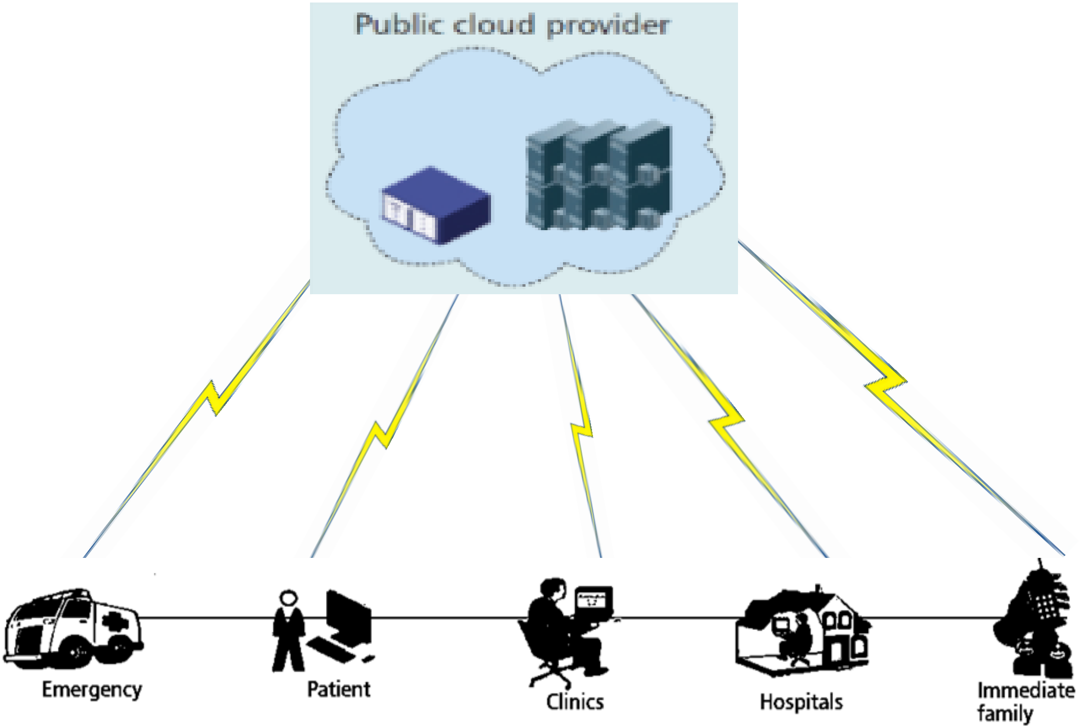
Public cloud provider
Emergency
Patient
Clinics
Hospitals
Immediate family

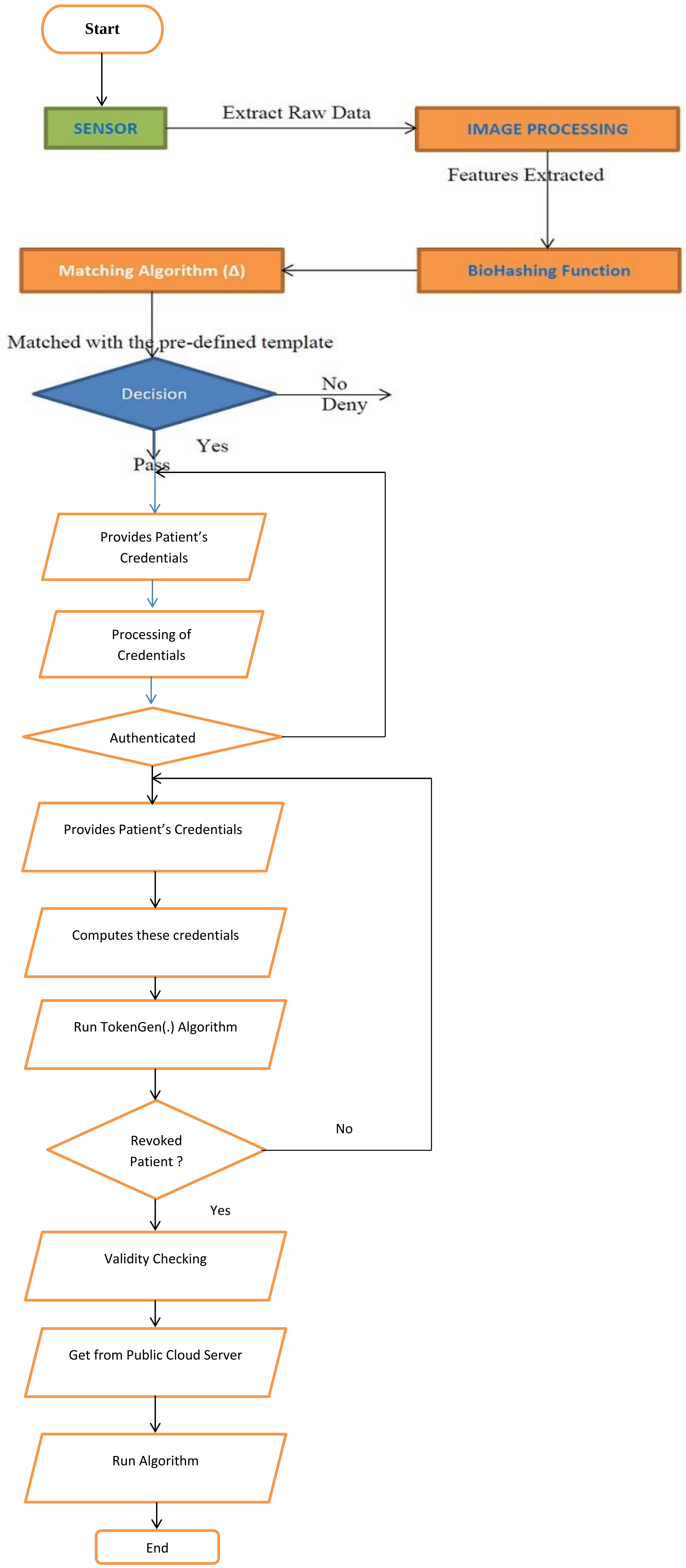

Start
SENSOR
Extract Raw Data
IMAGE PROCESSING
Features Extracted
BioHashing Function
Matching Algorithm (Δ)
Matched with the pre-defined template
Decision
No
Deny
Yes
Pass
Provides Patient's Credentials
Processing of Credentials
Authenticated
Provides Patient's Credentials
Computes these credentials
Run TokenGen(.) Algorithm
Revoked Patient ?
No
Yes
Validity Checking
Get from Public Cloud Server
Run Algorithm
End

User (U)	Public Cloud Server (PCS)
**AUTHENTICATION PHASE**	
Enter: ID_P, PW_P<br>Generate: B_P<br>Pick: r_{P2} G_P from the memory<br>Calculate: $\alpha_P=Rep(B_P, \beta_P)$<br>$r_{P1}=r_{P2}\oplus h(\alpha_P)$<br>$HPW_P=h(ID_P\|\|r_{P1}\|\|PW_P)$<br>$Q_P=HPW_P\oplus G_P$<br>$C_P^{/}=h(ID_P\|\|Q_P\|\|HPW_P)$<br>Confirm: $C_P^{/}?=C_P$<br>Pick: $a\in Z^{*}_{q}$<br>Calculate: $W_P=a.O$<br>$V_P=h(ID_P\|\|Q_P\|\|W_P\|\|T_1)$	
$\{W_P, V_P, CID_P, T_1\}$ →	
	Confirm: T_1<br>Decrypt: CID_P *s*<br>$(ID_P\|\|r_{PCS})=D_s(CID_P)$<br>Compute: $Q_P=h(ID_P\|\|s)$<br>$V_P^{/}= h(ID_P\|\|Q_P\|\|W_P\|\|T_1)$<br>Confirm: $V_P^{/}?=V_P$<br>Pick: $b\in Z^{*}_{q}$,<br>Calculate: $X_P=b.O$<br>$SK_{PCS}=h(ID_P\|\|Q_P\|\|X_P\|\|T_1)$<br>Pick: $r_{PCS}^{new}\in Z^{*}_{q}$<br>Compute: $CID_P^{new}=E_s(ID_P\|\|r_{PCS}^{new})$<br>$V_P=h(Q_P\|\|CID_P^{new}\|\|SK_{PCS}\|\|T_2)$
← $\{X_P, V_P, CID_P^{new}, T_2\}$	
Confirm: T_2<br>Calculate: $SK_P=h(ID_P\|\|Q_P\|\|W_P\|\|T_1)$<br>$V_P=h(Q_P\|\|CID_P^{new}\|\|SK_P\|\|T_2)$<br>Confirm $V_P?=V_P$ and replace CID_P with CID_P^{new}	

PASSWORD CHANGE PHASE

Enter Old identity: ID_P

Enter Old Password: PW_P

Imprint Biometrics: B_P

Calculate: $\alpha_P=Rep(B_P, \beta_P)$

$r_{P1}=r_{P2}\oplus h(\alpha_P)$

$HPW_P=h(ID_P||r_{P1}||PW_P)$

$Q_P=HPW_P\oplus G_P$

Verify: $C_P?=h(ID_P||Q_P||HPW_P$

Enter New Password: PW_P^{new}

Generate New Biometrics B_P^{new}

Extracts: r_P^{new}

Compute: $Gen(B_P^{new})=(\alpha_P^{new},\beta_P^{new})$

$HPW_P^{new}=h(ID_P||r_P^{new}||PW_P^{new})$

$G_P^{new}=HPW_P^{new}\oplus Q_P$

$C_P^{new}=h(ID_P||Q_P||HPW_P^{new})$

$r_{P1}=r_{P2}\oplus h(\alpha_P^{new})$

Update: $\{r_{P1}, C_P, r_{P2}, \beta_P\}$ with $\{r_{P1}^{new}, C_P^{new}, r_{P2}^{new}, \beta_P^{new}\}$

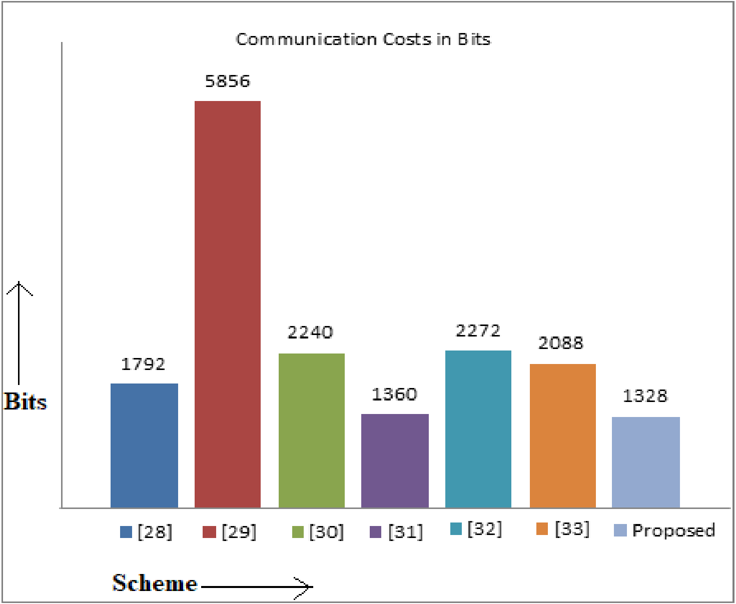
Communication Costs in Bits
1792
5856
2240
1360
2272
2088
1328
Bits
[28]
[29]
[30]
[31]
[32]
[33]
Proposed
Scheme

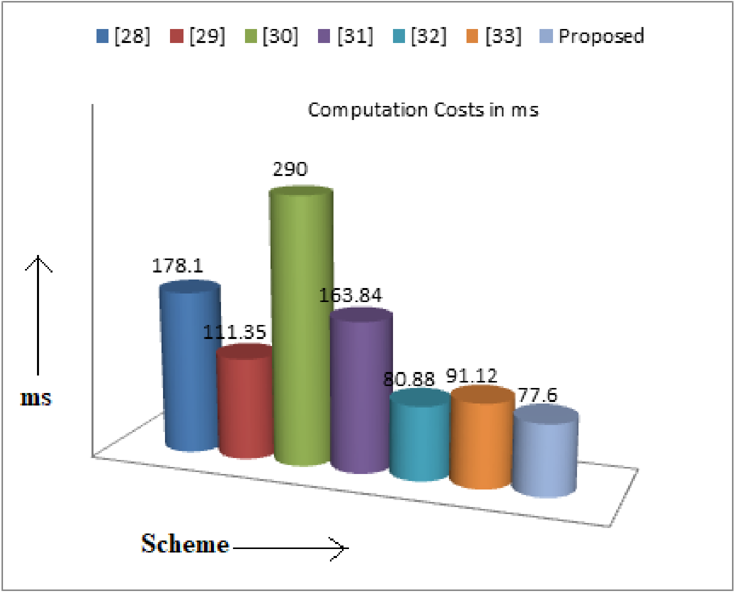
[28]
[29]
[30]
[31]
[32]
[33]
Proposed
Computation Costs in ms
178.1
111.35
290
163.84
80.88
91.12
77.6
ms
Scheme
