# Supplementary material for: A lightweight and robust authentication scheme for the healthcare system using public cloud server
Source: PLoS One. 2024 Jan 30;19(1):e0294429. doi: 10.1371/journal.pone.0294429 (PMC10826970; doi:10.1371/journal.pone.0294429)
Supplement: S2 File — (DOCX) [file pone.0294429.s003.docx]

**
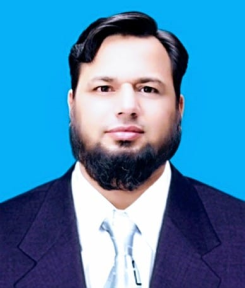
IRSHAD AHMED ABBASI (MEMBER, IEEE**) received the M.S. degree in computer science from COMSATS University Islamabad, Abbottabad Campus, Pakistan, in 2011 and the Ph.D. degree in computer science from Universiti Malaysia Sarawak, Malaysia, in 2019. He worked as a Senior Lecturer at King Khalid University, Saudi Arabia, from 2011 to 2015. He worked as a Postdoctoral Research Fellow at Universiti Malaysia Sarawak. He is working as an Assistant Professor with the Department of Computer Science, University of Bisha, Saudi Arabia. He has over 12 years of research and teaching experience. He is the author of many articles published in top quality journals. His research interests include networks, VANETs, MANETs, FANETs, mobile computing, the IoT, cloud computing, cybersecurity, cryptography, soft computing, and drone security and authentication. He has received multiple awards, scholarships, and research grants. He is serving as an editor. He is also acting as a reviewer for many well reputed peer-reviewed international journals and conferences.

**
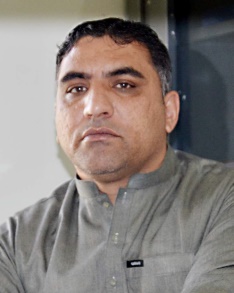
SAEED ULLAH JAN** received doctorate from University of Malakand in 2021. He is working an Assistant Provfessor of Computer Science at Higher Education, Achieves & Libraries Department Govt of Khyber Pakhtunkhwa – Pakistan. He is also working as Principal for the newly etsablished Govt College Darora (Dir Upper) – a far-flung remote area of the province where most of the youngsters have no access to Universities/Institutions for Higher Education. Furthermore, he has conducted research in many areas, including Information Security, Cloud Computing, Distributed Computing, Privacy-Preserving Parallel Computation, and Drone Security & Authentication. He has published over 25 research articles in prestigious conferences and journals and written an introductory Book in Computer Science for beginners. The Government of Khyber Pakhtunkhwa, Pakistan awarded him the “Best Teacher Award" for the year 2019-20 out of 11000 College Teachers in 309 public sector colleges in the Province.

**
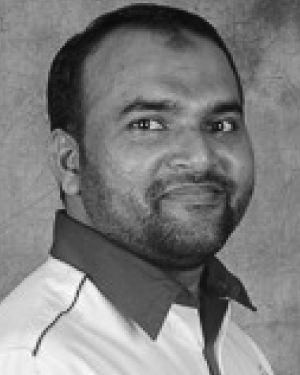
ADNAN SHAHID KHAN (SENIOR MEMBER, IEEE**) received the B.Sc. degree (Hons.) in computer science from the University of the Punjab, Lahore, Pakistan, in 2005, and the master’s, Ph.D., and Postdoctoral degrees in networks and information security from the Universiti Technology Malaysia, Johor Bahru, Malaysia, in 2008, 2012, and 2013, respectively. He is currently an Associate Professor with the Faculty of Computer Science and Information Technology, Universiti Malaysia Sarawak (UNIMAS). His research interests include cybersecurity in wireless communication, cloud computing, the Internet of Things, software-defined networking, cryptography, networks, and information security.

**
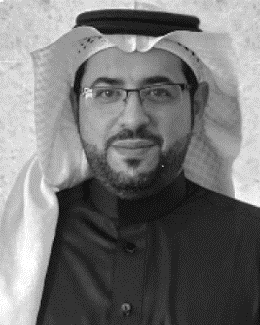
**

**FAHAD ALGARNI** received the bachelor's (First Class Hons.) degree in computer sciences from the Department of Computer Science, King Abdul-Aziz University, Jeddah, Saudi Arabia, in 2004, the master's degree in information technology (computer networks) from La Trobe University, Melbourne, VIC, Australia, in 2009, and the Ph.D. degree in information technology from the Clayton School of Information Technology, Monash University, Melbourne, VIC, Australia, in 2015. He is currently the Dean of the College of Computing and Information Technology, University of Bisha, Bisha, Saudi Arabia. His research interests include wireless sensor networks, cloud computing, systems’ reliability, Internet of Things, and cybersecurity

**
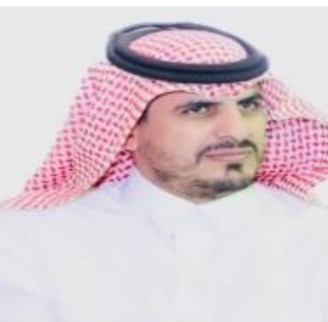
ABDULRAHMAN SAAD ALQAHTANI**Currently working as Associate Professor, College of Computing and Information Technology, Department of Computer Science, Bisha University, Kingdom of Saudi Arabia. His research interest includes software defined networks, block chain technology, networking, routing, wireless networks mobile computing, IoT, cloud computing, security and deep learning. He is a member of various Academic committees, External Bodies and Research Divisions. He is guiding the Research Students in the area of Information and Communication Engineering at Different Universities. He is also editor and reviewer of various national and international Journals. He is also an active member in various research societies related to his domain expertise and also having cordial contact with active researchers in those domains. In last fifteen years he has played a pivotal role in various technical symposiums and project Expo. He is also serving as a reviewer of many reputed journals and reviewed quality papers of peer researchers. He has also completed 2 new patents. He is also working on various international project funding agencies proposals to boost his research findings. Apart from this he continues his career as passionate Teacher and stay updated with current needs of IT industry and keeping himself updated and also taking active part in Industry-Academia collaborations in academics and research front.
