# Supplementary material for: A lightweight and robust authentication scheme for the healthcare system using public cloud server
Source: PLoS One. 2024 Jan 30;19(1):e0294429. doi: 10.1371/journal.pone.0294429 (PMC10826970; doi:10.1371/journal.pone.0294429)
Supplement: Attachment — Submitted filename: Response-to-Reviewers Comments.docx [file pone.0294429.s004.docx]

**JOURNAL REQUIREMENTS**

1. Please ensure that your manuscript meets PLOS ONE's style requirements, including those for file naming.

Response:	100% followed the journal format

2. Please note that PLOS ONE has specific guidelines on code sharing for submissions in which author-generated code underpins the findings in the manuscript.

Response:	We have mentioned our code in the Appendix A of the manuscript after references.

3. In your Data Availability statement, you have not specified where the minimal data set underlying the results described in your manuscript can be found.

Response:	The code is now mentioned in Appendix – A of the manuscript.

**EDITOR COMMENTS**

1) This paper has numerous sentence structure issues that need to be revised. For example:
   "...Despite the many advantages of cloud computing technologies for the healthcare industry, still, a variety of management, technological control, and regulatory issues are also to be taken into consideration, but two main issues, i.e., privacy and security of the current health system, are challenging for academia and industries..." Consider breaking down the above sentence in two or more sentences for ease of understanding.
2) The following sentence is incomplete: "..Because the healthcare system is gradually being converted to the public cloud environment in offering services to patients, researchers, labs, hospitals etc., in a flexible manner with minimal operational costs and in a reliable manner....
3) In the abstract, you need to describe the current solutions to public cloud server security issues. Thereafter, explain the challenges of these current solutions. This will help readers see the gap that this paper sought to fill.

**Author Response to comment 1-3: Dear Academic editor, please find herewith the revised abstract of the manuscript as per your valuable suggestions.**

4) Towards the end of the abstract, give a summary of the obtained results, preferably in terms of percentage improvements.

**Author Response: The said concern has been tackled in the exact section of the manuscript.**

5) "..A fuzzy extractor method is used to design the proposed protocol to make the authentication process more secure, and the system shows uniqueness while performing any task...."

i) Which task are you referring to?

ii) Which is this uniqueness that you are insinuating?

6) "...The proposed protocol is verifiably secure; this article has proved it using a well-known technique, i.e., BAN logic...."

You may state the above as follows:

"The BAN logic and ProVerif security proofs carried out demonstrate that the proposed protocol is verifiably secure"

A**uthor Response to comments 5-6: Yes, excellent for reminding, now we have revised the whole contribution statements in the manuscript.**

7) In the Literature Survey section, provide a critique of each of the current works discussed. This will help the readers appreciate the contributions of the proposed protocol.

**Author Response: Thanks, the whole section revised and critically review state of the art work.**

8) In "Review Analysis of Baseline Scheme", replace 'Baseline scheme' with the actual scheme that you are referring to. You may say, "Review Analysis of Azrour et al.'s scheme". The same should be done to "Weaknesses in Baseline Scheme"

**Author Response: Ok, Sir, thanks, revised as under:**

9) In section "Formal Security Analysis through Pragmatic Illustration", replace 'Pragmatic Illustration' with ' Random oracle model'.
10) In Section 5.4, "Informal Security Analysis through Discussion", remove 'through Discussion'

**Author Response to comments 9-10: These sections were renamed.**

11) Replace, "The parameters stored in the registration phase of the proposed scheme are the storage overheads" with "The parameters stored in the registration phase of the proposed scheme are used to calculate the storage overheads of this scheme"

**Author Response: Ok, Sir, thanks, revised as under:**

12) Include some future research scope in the conclusion section.
13) The conclusions must be drawn appropriately based on the data presented.

**Author Response to comments 12-13: Conclusion re-written along with the addition of future work**

14) The data should be provided as part of the manuscript or its supporting information, or deposited to a public repository.

**Author Response: The simulation code is given in appendix – A of the manuscript.**

**REVIEWER 1 COMMENTS**

**Dear Reviewer we are very grateful to you for your valuable concerns for improving our manuscript.**

1. Is the following point considered on the researcher’s contributions?: "An ECC-based lightweight authentication protocol has been proposed to securely provide services to the end-user in the cloud-enabled healthcare system"

**Author Response: Dear Reviewer, Yes, must be considered on the basis of the following reason:**

**a) ECC method consists of arithmetic encryption function, digital signature function, and verification function, which provide excellent security services to the patients while using cloud computing.**

**b) ECC Software Implementation is easily implemented and consumes less energy which means it is a lightweight asymmetric cryptographic approach.**

**c) The key is boundless security due to 160-bit in size**

**d) Scalar multiplication in ECC is not a time-consuming job.**

**e) The mathematical challenge in ECC based logarithms in finite fields of type integer Z_p_, which consist of integers modulo (a large prime p). Although this problem can be considered difficult, there are no known sub-exponential time algorithms for solving it.**

**f) In practical terms, sub-exponential time means that a determined hacker with enough processing power cannot break the system easily.**

2. The references used in Literature Survey are not arranged from oldest to newest. In addition to the limitations that some references suffered from, listed in Table 1, was the researcher able to overcome them? Please explain this in a separate paragraph within the section listed in Table 1

**Author Response: Thanks, we have revised the whole section; literature review section critically tackled.**

3. The references listed in Tables 7 and 8 are not included in the list of related works in Table 1.

**Author Response: Thanks, now, we have cited in the literature section of the paper.**

4. It is possible to use some numerical values for the communications costs of the proposed method in the conclusions. In addition to giving a future outlook for upcoming works in the same field

**Author Response: Ok, Sir, thanks, revised the conclusion, re-written and Futurework mentioned.**

5. Standardize the format of references. In addition to including at least two references published during the years 2022 and 2023

**Author Response: We have revised and modified by including 5 more references from the last two years.**

**REVIEWER 2 COMMENTS**

**Dear Reviewer we are very grateful to you for your valuable comments for improving our manuscript.**

1. In abstract, why low-complexity operations are required, the purpose has not been clarified?

**Author Response: The abstract of the article is revised.**

2. In abstract, it is not clear whether the security process is for authentication or for storing data in the cloud server.

**Author Response: Yes, great, Sir, thanks, now we have addressed this concern up to a maximum extent.**

3. In abstract, the limitations of previous work were not addressed.

**Author Response: Tackled in the revised version**

4. The introduction lacks references.

**Author Response: We have added more references as suggested**

5. The most closely related works obstacles to the proposed work were not explained in the introduction, nor were the mechanism of action of the proposed method to overcome them explained.

**Author Response: Enhanced the introduction in the modified version of the article. Yellow highlighted text shows that we have addressed this concern of the reviewer.**

6. Novelty is tinged with confusion.

**Author Response: At the end of the introduction section, contributions have been given, loopholes in previous work have been highlighted, and a scheme has been designed based on ECC using the Fuzzy Extractor method. The security analysis section has been tackled from four angles, i.e., BAN logic analysis, ROM analysis, ProVerif simulation, and threats discussion. At the same time, the performance metrics have been measured by considering storage, communication and computation costs. We have also compared our scheme with the state of the works regarding communication and computation costs.**

7. The “Related Work” section lacks of enough references. I strongly recommend that the author improve this section by adding references that support all the claims and motivation of the problem. The author may precisely and comprehensively point out the current issues and existing solutions. I suggest adding more related reference such as:
1- <https://ieeexplore.ieee.org/abstract/document/9842900>
2- <https://ieeexplore.ieee.org/abstract/document/7753621>
3- <https://www.mdpi.com/2076-3417/13/2/691>
4- <https://ieeexplore.ieee.org/abstract/document/9274321>

**Author Response: These papers have been cited.**

8. In related works section, the limitations of most works must be addressed and proven with a scientific argument or citation to a reference.

**Author Response: The related work section has been modified. Critical literature review section added.**

9. In “3.2 Elliptic Curve Cryptography (ECC)”, the last point is not clear.

**Author Response: Revise as under**

10. In 3.4 “Threat and Adversary Model”, the seventh point requires elaboration.

**Author Response: Elaborated**

11. In “Weaknesses in Baseline Scheme”, 4) Privileged-insider Attack, Proof is not enough.

**Author Response: Elaborated as shown below**

12. Identity in the registration process is transmitted openly and explicitly. This is a safe loophole.

**Author Response: The registration process is mainly accomplished over a private channel in offline mode, which is why open credentials don’t affect the security of a scheme.**

13. In “4.5 Patient Revocation/Re-registration Phase”, add flowchart explain this process.

**Author Response: Fig 2 has been given, which represent the whole scenario**

14. In security analysis, many attacks are not addressed, such as: forgery, known session key, Man-in-the-middle, replay, impersonation, Privileged insider and guessing, de-synchronization and DOS attacks.

**Author Response: The said attacks are defined, highlighted as yellow text in the manuscript**

15. The attacks in Table 8 must be security-analyzed and not just referred to in the table.

**Author Response: The attacks mentioned in table 8 have also been analyzed in the threats analysis section of the manuscript.**
