# Supplementary material for: A lightweight and robust authentication scheme for the healthcare system using public cloud server
Source: PLoS One. 2024 Jan 30;19(1):e0294429. doi: 10.1371/journal.pone.0294429 (PMC10826970; doi:10.1371/journal.pone.0294429)
Supplement: Attachment — Submitted filename: Rebuttal Letter(Response to Reviewers).docx [file pone.0294429.s005.docx]

**Rebuttal Letter**

**Dear Editor, We are very grateful to you for your valuable comments and suggestions that helped us to improve our manuscript. We have addressed all the concerns. Now, Paper is ready to be processed for publication.**

**EDITOR COMMENTS**

**Comment No. 01:** Please review your reference list to ensure that it is complete and correct. If you have cited papers that have been retracted, please include the rationale for doing so in the manuscript text, or remove these references and replace them with relevant current references. Any changes to the reference list should be mentioned in the rebuttal letter that accompanies your revised manuscript. If you need to cite a retracted article, indicate the article’s retracted status in the References list and also include a citation and full reference for the retraction notice.

**Author Response: It is to state that the previously cited papers were removed based on either old or irrelevant. After that, we have added new state–of––art and recent papers. Currently, we have added DOI to all papers and URL/Link to those papers without DOI.**

**Comment No. 2:** In the abstract, you need to describe the current solutions to public cloud server security issues. Thereafter, explain the challenges of these current solutions. This will help readers see the gap that this paper sought to fill.

**Author Response: Revised as directed. Yellow highlighted text inside abstract shows third round changes.**

**Comment No. 03:** "...The proposed protocol is verifiably secure; this article has proved it using a well-known technique, i.e.,BAN logic...."You may state the above as follows: "The BAN logic and ProVerif security proofs carried out demonstrate that the proposed protocol is verifiably secure"

Author Response: **Great, Sir, Thanks for correction – Revised as directed**

**Comment No. 04:** Avoid the usage of 'etc', 'i.e'. You have to write in full. Ensure that the conclusion section is one continuous paragraph.

**Author Response: Ok, Removed from the paper**

**Comment No. 05:** "...The proposed mechanism regarding storage overhead, communication, and computation cost is analyzed. "Replace with the following:"...The proposed mechanism is analyzed in terms of storage overhead, communication and computation costs...."

**Author Response: Addressed the said concern**

**Comment No. 06:** "...Upon comparing the proposed protocol with prior work in terms of security and performance metrics, it has been demonstrated that the proposed scheme is superior to its competitors...."Provide percentage improvements attained by your scheme in terms of storage, communication and computation costs.

**Author Response: Yes, done… Thanks**

**Comment No. 07:** "...A fuzzy extractor method is used to design the proposed protocol to make the authentication process more secure, and the system shows uniqueness while performing any task. Because an adversary cannot forge, extract, or collide the hash image generated from user biometrics in combination with a random key extracted before authentication...."Avoid starting your sentences with "Because...."

**Author Response: Revised as directed.**

**REVIEWERS’ COMMENTS**

**Comment No. 01:** If the authors have adequately addressed your comments raised in a previous round of review and you feel that this manuscript is now acceptable for publication, you may indicate that here to bypass the “Comments to the Author” section, enter your conflict of interest statement in the “Confidential to Editor” section, and submit your "Accept" recommendation.

**Reviewer #1:** All comments have been addressed

**Reviewer #2:** All comments have been addressed

**Author Response:** Thanks a lot……..!

**Comment No. 02:** Is the manuscript technically sounds, and do the data support the conclusions?

**Reviewer #1:** Yes

**Reviewer #2:** Yes

**Author Response:** Thanks

**Comment No. 03:** Has the statistical analysis been performed appropriately and rigorously?

**Reviewer #1:** Yes

**Reviewer #2:** Yes

**Author Response:** Obliged

**Comment No. 04:** Have the authors made all data underlying the findings in their manuscript fully available?

**Reviewer #1:** Yes

**Reviewer #2:** Yes

**Author Response:** Appreciations

**Comment No. 05:** Is the manuscript presented in an intelligible fashion and written in standard English?

**Reviewer #1:** Yes

**Reviewer #2:** Yes

**Author Response:** Thank you

**Comment No. 06:** Please use the space provided to explain your answers to the questions above. You may also include additional comments for the author, including concerns about dual publication, research ethics, or publication ethics. (Please upload your review as an attachment if it exceeds 20,000 characters)

**Reviewer #1:** (No Response)

**Reviewer #2:** All comments have been addressed and the authors answer all comments. I recommend accepting the article

**Author Response:** Appreciative

Regards.......!

Dr. Irshad Ahmed Abbasi.
